# Phylogenomic reappraisal of the family *Rhizobiaceae* at the genus and species levels, including the description of *Ectorhizobium quercum* gen. nov., sp. nov.

**DOI:** 10.3389/fmicb.2023.1207256

**Published:** 2023-08-03

**Authors:** Tengfei Ma, Han Xue, Chungen Piao, Ning Jiang, Yong Li

**Affiliations:** Key Laboratory of Forest Protection of National Forestry and Grassland Administration, Chinese Academy of Forestry, Beijing, China

**Keywords:** *Rhizobiaceae*, *Ectorhizobium*, cpAAI, UBCG, concatenated protein tree

## Abstract

The family *Rhizobiaceae* contains 19 validly described genera including the rhizobia groups, many of which are important nitrogen-fixing bacteria. Early classification of *Rhizobiaceae* relied heavily on the poorly resolved 16S rRNA genes and resulted in several taxonomic conflicts. Although several recent studies illustrated the taxonomic status of many members in the family *Rhizobiaceae*, several para- and polyphyletic genera still needed to be elucidated. The rapidly increasing number of genomes in *Rhizobiaceae* has allowed for a revision of the taxonomic identities of members in *Rhizobiaceae*. In this study, we performed analyses of genome-based phylogeny and phylogenomic metrics to review the relationships of 155-type strains within the family *Rhizobiaceae*. The UBCG and concatenated protein phylogenetic trees, constructed based on 92 core genes and concatenated alignment of 170 single-copy orthologous proteins, demonstrated that the taxonomic inconsistencies should be assigned to eight novel genera, and 22 species should be recombined. All these reclassifications were also confirmed by pairwise cpAAI values, which separated genera within the family *Rhizobiaceae* with a demarcation threshold of ~86%. In addition, along with the phenotypic and chemotaxonomic analyses, a novel strain BDR2-2^T^ belonging to a novel genus of the family *Rhizobiaceae* was also confirmed, for which the name *Ectorhizobium quercum* gen. nov., sp. nov. was proposed. The type strain is BDR2-2^T^ (=CFCC 16492^T^ = LMG 31717^T^).

## 1. Introduction

The family *Rhizobiaceae*, consisting of several “rhizobia” species and currently up to 19 genera and 181 species with validly described names (https://lpsn.dsmz.de/family/rhizobiaceae), was first proposed in 1938. Most of the members in the family *Rhizobiaceae* were widely known for their association with plant roots, including the induction of N_2_-fixing nodules (De Lajudie et al., 2019), tumors (Mousavi et al., 2014), or hairy roots (Jiang et al., 2019) on their host plants [e.g., *Rhizobium, Agrobacterium, Ensifer* (syn. *Sinorhizobium*), *Allorhizobium, Shinella, Neorhizobium*, and *Pararhizobium*].

The early taxonomic classification of *Rhizobiaceae* was circumscribed by two genera, *namely Rhizobium* and *Agrobacterium*, only based on nitrogen-fixing or pathogenic traits. As the phylogenetic analysis advances, many other novel genera belonging to the family *Rhizobiaceae* were proposed in succession based on the 16S rRNA phylogenetic trees [e.g., *Martelella* (Rivas et al., 2005), *Shinella* (An et al., 2006), *Ciceribacter* (Kathiravan et al., 2013), *Lentilitoribacter* (Park et al., 2013), *Liberibacter* (Fagen et al., 2014), and *Gellertiella* (Tóth et al., 2017)]. Over time, the phylogenetic method of multilocus sequence analysis (MLSA), which provides a more robust taxonomic resolution (De Lajudie et al., 2019), was used in the revision of the genera within *Rhizobiaceae*, and the proposal of several novel genera and combinations made the classifications of *Rhizobiaceae* more precise (Ramírez-Bahena et al., 2008; Kimes et al., 2015; Rocha et al., 2020). At present, the defined prokaryotic genera or higher taxa relies heavily on the monophyly of species (De Lajudie et al., 2019), and the genome-based phylogeny was considered to be a more convenient and accurate method (Parks et al., 2018). Benefiting from the advances in next-generation sequencing technology, an enormous amount of genomic data had been accumulated in public databases, which provided the base for a more accurate classification of prokaryotes. Based on the genomic data of the family *Rhizobiaceae* in the public databases, the genus *Ciceribacter* (Rahi et al., 2021), Agrobacterium *tumefaciens* species complex G3 (Singh et al., 2021), and *Rhizobium leguminosarum* species complex (Young et al., 2021) were revised, along with various genomic metrics, and several new species combinations were proposed. Recently, a genomic metric of cpAAI data was proposed to define the genera in the family *Rhizobiaceae* with a threshold of ~86%, and several new genera and combinations were proposed (Kuzmanović et al., 2022). However, there are several taxonomic inconsistencies within *Rhizobiaceae* that need to be elucidated.

During our study of the bacterial diversity in the disease of oaks, the strain BDR2-2^T^ was isolated from the symptomatic bark of *Quercus acutissima* caker. Preliminary phylogeny analysis showed that the strain BDR2-2^T^ should be assigned to the family *Rhizobiaceae*. In this study, we combined the UBCG and 120 ubiquitous single-copy protein phylogenetic analyses, along with the genomic metrics of AAI, POCP, and cpAAI, to confirm the taxonomic status of BDR2-2^T^ and other conflicts of species within the family *Rhizobiaceae*.

## 2. Materials and methods

### 2.1. Strain and culture conditions

The strain BDR2-2^T^ was isolated from the symptomatic bark of *Q. acutissima* caker collected from Hefei, China (31°50′28″N, 117°10′34″E). The isolation and purification of the strain BDR2-2^T^ were performed as previously described (Ma et al., 2022). In brief, the samples were initially surface-sterilized successively in 70% ethanol for 30 s and 4% (v/v) sodium hypochlorite for 2 min. After washing with sterile water for three times, the samples were transferred to a sterile mortar, ground with a pestle, and then cultivated for 30 min. The suspensions were spread on yeast extract mannitol agar (YMA) with a dilution series. After 2 days of incubation at 30°C, single colonies were cultured on a new plate and then preserved at −80°C.

### 2.2. Genome sequencing and reference genome

The genome of the strain BDR2-2^T^ was sequenced with Illumina NovaSeq PE150 by Novogene, Co., Ltd. (Beijing, China). In brief, the low-quality reads were filtered by readfq (version 10), and then, the high-quality reads were assembled using SOAPdenovo (version 2.04) (Li et al., 2008, 2010), SPAdes (Bankevich et al., 2012), and ABySS (Simpson et al., 2009). After integrating with CISA (Lin and Liao, 2013), the gaps in the results were filled with gapclose (version 1.12). In this study, 136 validated *Rhizobiaceae* and 18 unvalidated *Rhizobiaceae* were analyzed. Because the 16S rRNA phylogenetic tree was inappropriate for delineating genera in the family *Rhizobiaceae* in previous studies (e.g., strains from *Brucellaceae* nested in *Rhizobiaceae*) (Hördt et al., 2020), five type strains from *Brucellaceae* (including the type genus *Brucella* and type species from the other two genera) were also analyzed in this study to confirm it. In addition, five strains from *Caulobacterales* were also used as an out-group in this study. The type strain genome sequences used were obtained from the NCBI database, and all of the genome sequences were assessed by CheckM (Parks et al., 2015).

### 2.3. Phylogenetic analyses

Full-length 16S rRNA gene sequences were extracted from the genomes via RNAmmer 1.2 for the phylogenetic analysis (Lagesen et al., 2007). The multiple alignments of the sequences were performed with Clustal W, and then, the phylogenetic trees were constructed with MEGA X by the methods of maximum-likelihood, neighbor-joining, and maximum-parsimony (Kumar et al., 2018). The phylogenetic trees were evaluated by 1,000 bootstrap resamplings, and the species of *Brucellaceae* and *Caulobacterales* were used as the out-group.

A phylogenomic tree, particularly a concatenated core gene tree, was considered to be a more convenient and accurate substitute method for taxonomic analysis as it provides a higher resolution phylogeny (Kim et al., 2021). There are 92 core genes that were extracted from the genomes using the command “java -jar UBCG.jar extract” and used in the UBCG phylogenetic tree, which was generated by RAxML using the command “jar -jar UBCG.jar align.” The species of *Brucellaceae* and *Caulobacterales* were used as the out-group.

Additionally, another phylogenomic tree with a concatenated alignment of 170 ubiquitous single-copy proteins was constructed with FastTree. The extraction and alignment of the sequences were generated with the method at github.com/flass/cpAAI_Rhizobiaceae (Kuzmanović et al., 2022), and the tree was visualized and edited with iTOL (Letunic and Bork, 2021).

### 2.4. Genome-based metrics analyses

For the species level, average nucleotide identity (ANI) and genome-to-genome distance comparison (GGDC) are currently two standard practices for species delineation (Goris et al., 2007; Richter and Rosselló-Móra, 2009), and the values of ANI and dDDH were determined with pyani (Pritchard et al., 2016) and genome-to-genome distance comparison (GGDC, http://ggdc.dsmz.de), respectively. For higher taxonomic ranks, core-proteome average amino acid identity (cpAAI) has recently been proposed for genus delineation within *Rhizobiaceae* (Kuzmanović et al., 2022), and the pairwise cpAAI values within *Rhizobiaceae* were calculated by the cpAAI_Rhizobiaceae code (https://github.com/flass/cpAAI_Rhizobiaceae). The percentage of conserved proteins (POCPs) and average amino acid identity (AAI) were two standardized metrics for genus-level delineation. A Python script (POCP, https://github.com/2015qyliang/POCP) was used to calculate POCP, while CompareM (https://github.com/dparks1134/CompareM) was used for AAI (Ma et al., 2022).

### 2.5. Chemotaxonomy and physiology

The polar lipids and isoprenoid quinones were performed as described by Minnikin et al. (1984) and Collins et al. (1977), respectively. The extraction of cellular fatty acids was performed as described by Kuykendall et al. (1988) and then analyzed with the Sherlock Microbial Identification System (MIDI) (Sasser, 1990). The growth gradients of pH, temperature, and salinity were optimized by the methods described by Li et al. (2016). Gram staining was carried out as described by Jenkins et al. (2003). The test of anaerobic growth was performed in an anaerobic jar for a week (Li et al., 2016). The activities of oxidase and catalase were determined by the methods described by Li et al. (2016). Enzymatic activity, acid production, and carbon source utilization were performed using API ZYM, API 50CH, and API 20NE (bioMérieux) according to the manufacturer's instructions.

## 3. Results and discussion

**Figure 1:**
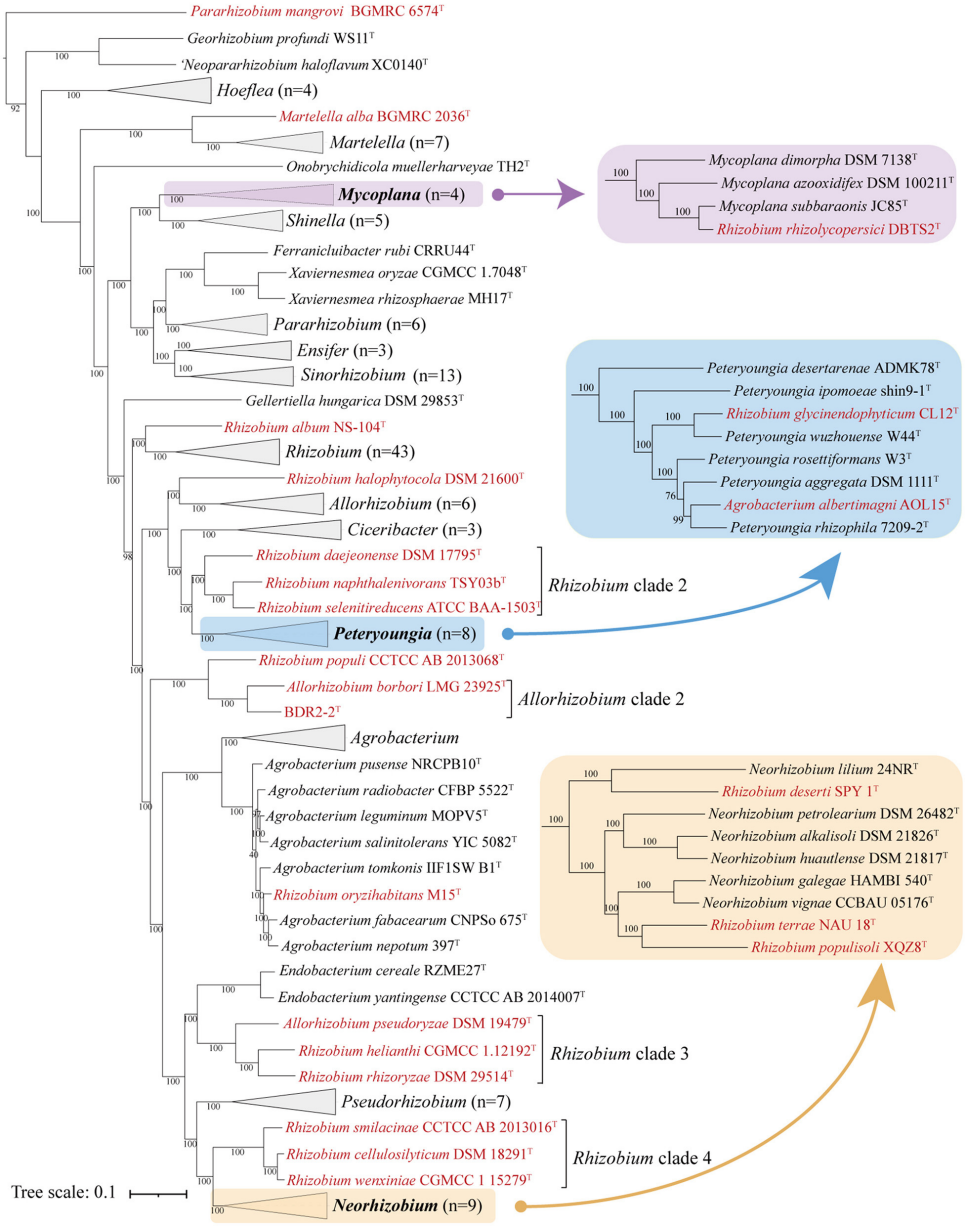
Concatenated protein phylogenetic tree among strains in the family *Rhizobiaceae* based on a concatenated alignment of 170 ubiquitous single-copy proteins. For details and abbreviations see Supplementary Figure S2. The scale bar corresponds to 0.1 substitutions per amino acid position.

The 16S rRNA gene phylogeny was widely used in prokaryote taxonomic analyses due to its high conservation (Park et al., 2013; Fagen et al., 2014; Tóth et al., 2017), and this, on the other hand, generally did not provide sufficient resolution for closely related species (Vinuesa et al., 2005; Liang et al., 2021). As expected, the full-length 16S rRNA phylogenetic tree showed low bootstrap support at the genus and species levels, resulting in poorly resolved taxonomic issues (Supplementary Figure S1). The concatenated proteins and UBCG trees showed a similar phylogenetic backbone to each other, and most of the species in the family *Rhizobiaceae* consistently grouped into similar monophyletic clades with high bootstrap values. The concatenated protein is shown in Figure 1, and the full details of the two phylogenetic trees are shown in Supplementary Figures S2, S3. For genus demarcation within *Rhizobiaceae*, the genomic metric of cpAAI data was recently proposed, with a threshold of ~86% (Kuzmanović et al., 2022), and here we calculated the pairwise cpAAI values to confirm the reclassification of the *Rhizobiaceae* order.

**Figure 2:**
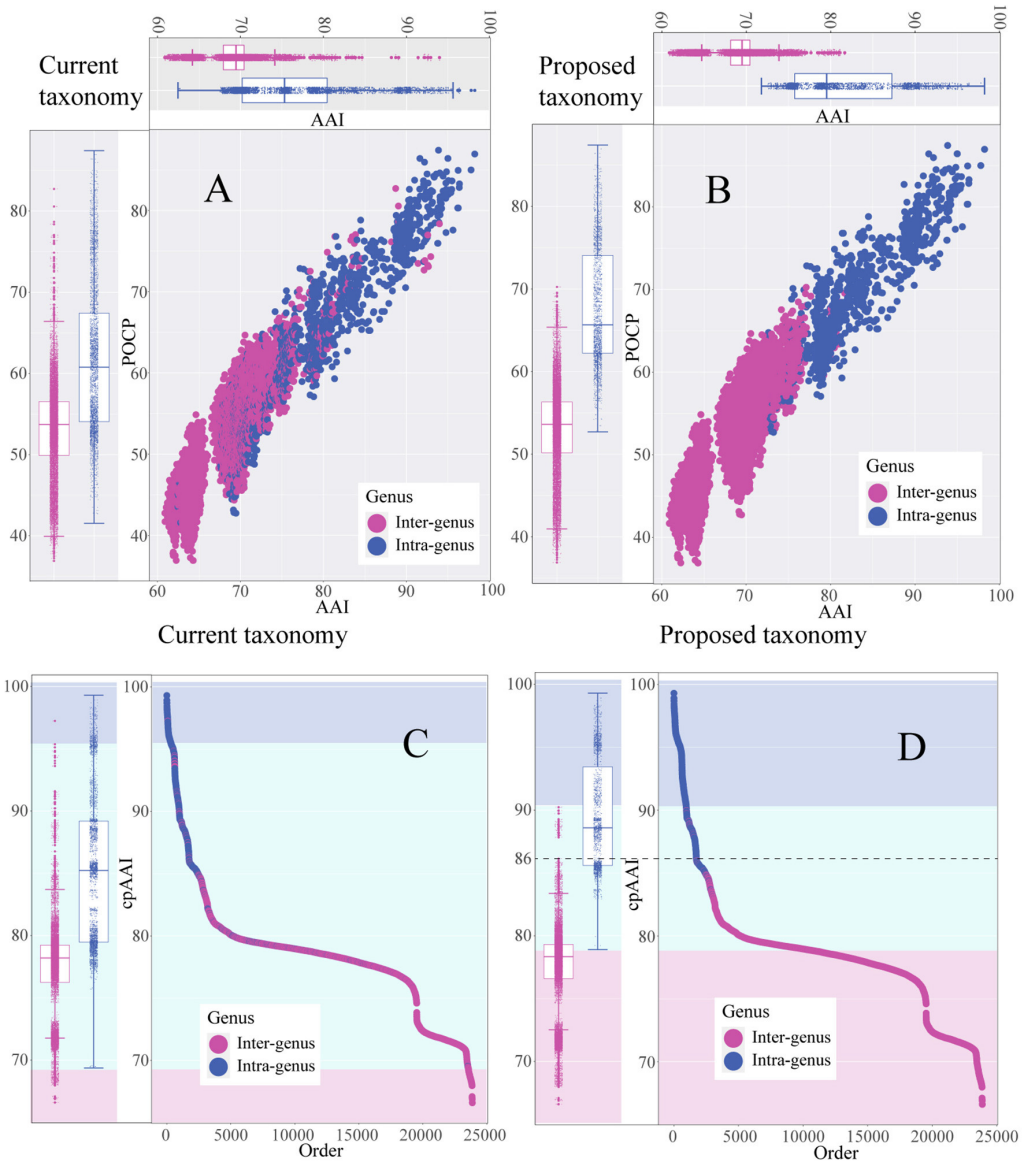
Scatter plot and the box plot represent the distribution of pairwise average amino acid identity (AAI) values, percentage of conserved protein (POCP) values, and core-proteome average amino acid identity (cpAAI) within and between the genera of the family *Rhizobiaceae*. **(A, B)** Represent pairwise AAI and POCP values of the current genera and the proposed genera, respectively. **(C, D)** Represent the rank order of pairwise cpAAI values of the current genera and the proposed genera, respectively. The background color light blue represents the proposed genus, light cyan represents closely related to the proposed genus, and light red represents distantly related to the proposed genus.

As shown in the pairwise AAI, POCP, and cpAAI values of the currently proposed genera in the family *Rhizobiaceae*, the pairwise values between inter-genus and intra-genus could not be separated (Figures 2A, C), and there should be several misclassification species among the currently proposed genera. By applying the cpAAI threshold of ~86% for genus demarcation and combining it with phylogenetic tree analysis, most of the pairwise values could be clearly separated between inter-genus and intra-genus (Figures 2B, D). The pairwise AAI, POCP, and cpAAI values are shown in Supplementary Table S1.

### 3.1. Reclassification of *Rhizobiaceae* at the genus level

All phylogenetic trees (Figure 1, Supplementary Figures S2, S3) showed that most genera within the family *Rhizobiaceae* were clustered into monophyletic clades, except for several genera that formed paraphyletic or polyphyletic clades. Among those paraphyletic or polyphyletic clades, the taxonomic conflicts were resolved as follows. All reclassifications were also confirmed by the genus demarcation of cpAAI with a threshold of ~86%.

As for the paraphyletic genus, which consisted of a monophyletic clade with one or more species of a different genus (Wood, 1994; Liang et al., 2021), the conflicting clade should be merged into the primary genus. *Martelella* appeared like a paraphyletic genus in both phylogenetic trees because *Martelella alba* BGMRC 2036^T^, a recently proposed novel species, formed an outermost clade of the *Martelella* lingage by a long branch, and *M. alba* BGMRC 2036^T^ might be a different genus from *Martelella*. Furthermore, the pairwise cpAAI values between *M. alba* BGMRC 2036^T^ and other *Martelella* strains ranged from 79.5 to 80.5% (Supplementary Table S1 and Supplementary Figure S4A), which were also significantly lower than the recommended genus demarcation value of 86%, and therefore, we proposed to transfer *M. alba* BGMRC 2036^T^ to a novel genus *Paramartelella* gen. nov.

*Mycoplana* was shown as paraphyletic in all phylogenetic trees (Figure 1, Supplementary Figures S2, S3) because *Rhizobium rhizolycopersici* DBTS2^T^ was nested within *Mycoplana* with high support. In the original proposal of *R. rhizolycopersici* DBTS2^T^, the phylogenetic tree was constructed with a low number of closely related taxa, and the *Mycoplana*-type strain was not considered (Thin et al., 2021). Therefore, we proposed to assign *R. rhizolycopersici* DBTS2^T^ to *Mycoplana*. In addition, the pairwise cpAAI values within the *Mycoplana* clade ranged from 89.7 to 97.2% (Supplementary Table S1 and Supplementary Figure S4B), and those values were also higher than the genus demarcation threshold, which further confirmed the classification.

Similarly, the genera *Peteryoungia, Agrobacterium, Neorhizobium* also appeared as paraphyletic in all phylogenetic trees (Figure 1, Supplementary Figures S2, S3). *Rhizobium glycinendophyticum* CL12^T^ and *Agrobacterium albertimagni* AOL15^T^ were nested within *Peteryoungia* with a high bootstrap value. The pairwise cpAAI values between the two strains and other *Peteryoungia* strains were also higher than the genus demarcation threshold (Supplementary Table S1 and Supplementary Figure S4C), which confirmed that *R. glycinendophyticum* CL12^T^ and *A. albertimagni* AOL15^T^ should be transferred to *Peteryoungia*. Using a similar method as above, *Rhizobium oryzihabitans* M15^T^ was nested within *Agrobacterium*, and *Rhizobium deserti* SPY 1^T^, *Rhizobium populisoli* XQZ8^T^, and *Rhizobium terrae* NAU 18^T^ were nested within *Neorhizobium*. Pairwise cpAAI values confirmed that *R. oryzihabitans* M15^T^ should be assigned to *Agrobacterium* (Supplementary Table S1 and Supplementary Figure S4D), and *R. deserti* SPY 1^T^, *R. populisoli* XQZ8^T^, and *R. terrae* NAU 18^T^ should be assigned to *Neorhizobium* (Supplementary Table S1 and Supplementary Figure S4E).

Different from the paraphyletic genus, the polyphyletic genus was typically more difficult to resolve, as the taxonomic issues were done by merging the conflicting clades or transferring them to novel genera (Farris, 1974; Liang et al., 2021). *Pararhizobium* appeared as polyphyletic in the phylogenetic trees (Figure 1, Supplementary Figures S2, S3) because *Pararhizobium mangrovi* BGMRC 6574^T^ and *Pararhizobium haloflavum* XC0140^T^ were placed in a distant position relative to the genus *Pararhizobium* with high support values. This analysis confirmed *P. haloflavum* XC0140^T^, which was not validly published despite being proposed as “*Neopararhizobium*” (Hördt et al., 2020) and represents a novel genus. *Pararhizobium mangrovi* BGMRC 6574^T^, a recently proposed novel species, formed the outermost clade of “*Neopararhizobium”* and *Georhizobium* lineages with a distant evolutionary relationship with *Pararhizobium* in all phylogenetic trees, which implied that the strain should be assigned to a novel genus. The pairwise cpAAI values between *P. mangrovi* BGMRC 6574^T^ and other *Pararhizobium* strains ranged from 69.4 to 69.5%, which were also significantly lower than the recommended genus demarcation value of 86%, and therefore, we proposed to transfer *P. mangrovi* BGMRC 6574^T^ to a novel genus *Allopararhizobium* gen. nov.

Although most taxonomic conflicts of the genus *Rhizobium* were resolved, *Rhizobium* was shown as polyphyletic in the phylogenetic trees (Figure 1, Supplementary Figures S2, S3), including strains such as *Rhizobium album* NS-104^T^, *Rhizobium halophytocola* DSM 21600^T^, *Rhizobium* clade 2, *Rhizobium populi* CCTCC AB 2013068^T^, *Rhizobium* clade 3, and *Rhizobium* clade 4, which were placed apart from the genus *Rhizobium*. *Rhizobium album* NS104^T^ formed the outermost clade of the genus *Rhizobium* lineage in all phylogenetic trees and showed a distant evolutionary relationship with other *Rhizobium* species, indicating that the sole species might represent a novel genus in the family *Rhizobiaceae*. In addition, the pairwise cpAAI values between *R. album* NS104^T^ and other *Rhizobium* strains ranged from 80.6 to 82.3% (Supplementary Table S1 and Supplementary Figure S4F), which were also significantly lower than the recommended genus demarcation value, and therefore *R. album* NS104^T^ represented a novel genus *Metarhizobium* gen. nov.

**Figure 3:**
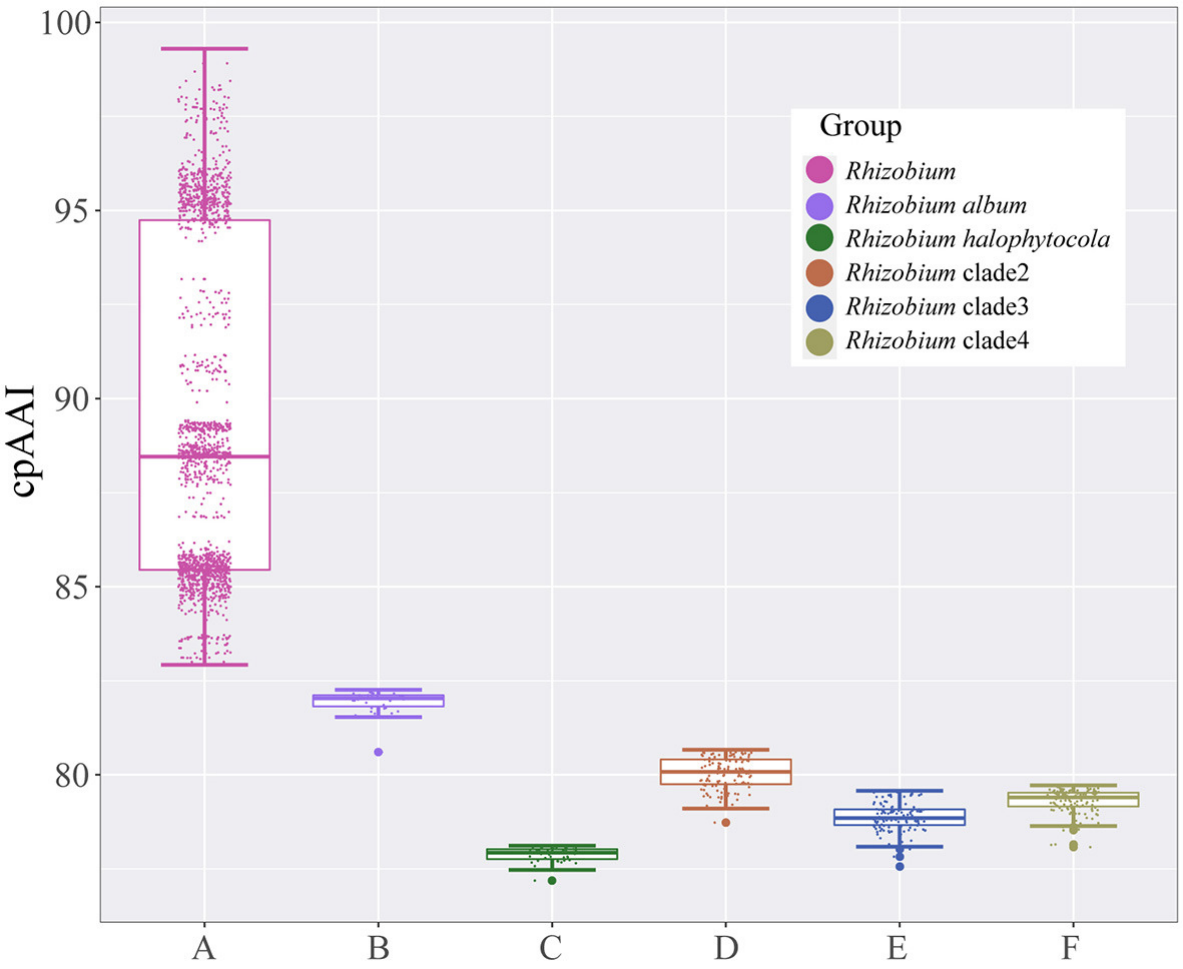
Box plot of pairwise core-proteome average amino acid identity (cpAAI) values within the genus *Rhizobium* and between genus *Rhizobium* and other *Rhizobium* clades. A represents the values within the genus *Rhizobium*; B–F represent the values between *Rhizobium album, Rhizobium halophytocola, Rhizobium* clade 2, *Rhizobium* clade 3, *Rhizobium* clade 4, and genus *Rhizobium*, respectively.

*Rhizobium halophytocola* DSM 21600^T^, *Rhizobium* clade 2, *Rhizobium* clade 3, and *Rhizobium* clade 4, which formed four different highly supported monophyletic clades, were placed apart from the *Rhizobium* lineage in the phylogenetic trees and separated from the species in the genus *Rhizobium*, implying that they should be transferred to four different novel genera. With the similar analytical methods as above, the pairwise cpAAI values also confirmed that *R. halophytocola* DSM 21600^T^ and *Rhizobium* clades 2–4 should belong to four different novel genera in the family *Rhizobiaceae* (Supplementary Table S1 and Supplementary Figures S5G–J). The pairwise cpAAI values within the *Rhizobium* lineage were also significantly higher than these values between *R. album* NS104^T^, *R. halophytocola* DSM 21600^T^, *Rhizobium* clades 2–4, and *Rhizobium* lineage (Figure 3), which also confirmed that these clades belong to five different novel genera.

In addition, the physiological and chemotaxonomic features could also distinguish these proposed novel genera from the *Rhizobium*-type strain (*R. leguminosarum* USDA 2370^T^). *R. albu*m NS-104^T^ grows over a pH range of 5.0–9.0 (optimum, 6.0), and *R. leguminosarum* USDA 2370^T^ grows at a pH range of 6.0–8.0 (optimum, pH 7.0–7.5) (Ramírez-Bahena et al., 2008; Hang et al., 2019). The growth of *R. halophytocola*v DSM 21600^T^ was observed up to 7.5% (w/v) NaCl (optimum, 4–5%); however, no growth of *R. leguminosarum* USDA 2370^T^ was observed in the presence of 1% NaCl (Ramírez-Bahena et al., 2008; Bibi et al., 2012). The percentages of major cellular fatty acids in *Rhizobium* clade 2 (above 67.8%) were also significantly different from *R. leguminosarum* USDA 2370^T^ (57.2%) (Tighe et al., 2000; Quan et al., 2005; Kaiya et al., 2012). *Rhizobium* clade 3 could grow at 40°C, at a pH range of 5.0–11.0, and at NaCl concentrations up to 4% (w/v) NaCl (optimum, 1%), which could distinguish *Rhizobium* clade 3 from *R. leguminosarum* USDA 2370^T^ (Zhang G. X. et al., 2011; Zhang X. X. et al., 2014; Wei et al., 2015). The assimilation of L-malate, L-arabinose, gluconate, and the amount of C_16:0_ and summed feature 2 (C_12:0_ aldehyde and/or unknown 10.928) also separate *Rhizobium* clade 4 from *R. leguminosarum* USDA 2370^T^ (Garcia-Fraile et al., 2007; Zhang L. et al., 2014; Gao et al., 2017).

Altogether, *R. halophytocola* DSM 21600^T^ should be assigned to a novel genus *Heterorhizobium* gen. nov. *Rhizobium* clade 2 should belong to a novel genus *Paenirhizobium* gen. nov., with *Paenirhizobium daejeonense* comb. nov. as the type species. *Rhizobium* clade 3 should be assigned to *Affinirhizobium* gen. nov., with *Affinirhizobium pseudoryzae* comb. nov. as the type species. *Rhizobium* clade 4 should be assigned to *Alirhizobium* gen. nov., with *Alirhizobium cellulosilyticum* comb. nov. as the type species.

### 3.2. Proposal for *Ectorhizobium quercum* gen. nov., sp. nov.

#### 3.2.1. Genome-based phylogenetic analyses

**Figure 4:**
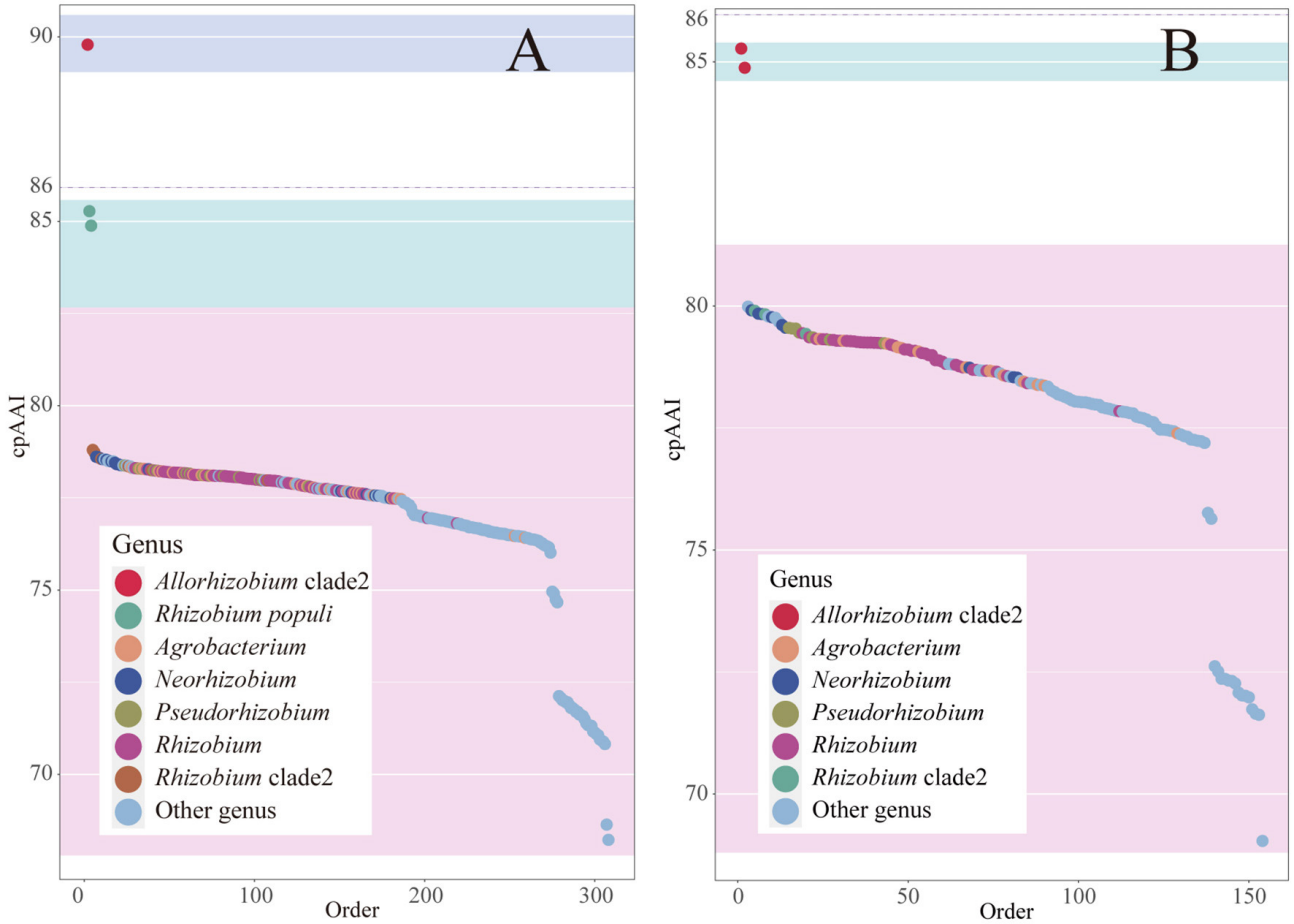
Rank order of pairwise cpAAI within the *Allorhizobium* clade and between the clade and other members within the family *Rhizobiaceae* is shown in plot **(A)**. The rank order of pairwise cpAAI between *Rhizobium populi* K-38^T^ and other members within the family *Rhizobiaceae* is shown in plot **(B)**. The background color light blue represents the proposed genus, light cyan represents closely related to the proposed genus, and light red represents distantly related to the proposed genus.

The 16S rRNA sequence pairwise comparisons showed that the strain BDR2-2^T^ was most closely related to *Allorhizobium borbori* DN316^T^ (97.4% similarity), followed by *R. populi* K-38^T^ (96.9% similarity), and less than 96.5% similarity with other species of the family *Rhizobiaceae* in the EzBioCloud database (Yoon et al., 2017), and therefore, the strain BDR2-2^T^ might belong to a novel species of *Rhizobiaceae*. The strain BDR2-2^T^, *A. borbori* DN316^T^, and *R. populi* K-38^T^ consistently formed a highly supported monophyletic lineage closer to *Gellertiella hungarica* DSM 29853^T^ or other lineages than to the genus *Allorhizobium* and *Rhizobium* lineages (Figures 1, 4), indicating that the three strains should be allocated to novel *Rhizobiaceae* genera. The cpAAI values between BDR2-2^T^ and *A. borbori* DN316^T^ were significantly higher than the genus demarcation threshold, indicating that *Allorhizobium* clade 2 should belong to the same genus. The pairwise cpAAI values between *R. populi* K-38^T^ and *Allorhizobium* clade 2 were 84.9 and 85.3%, respectively (Figure 4), which were slightly lower than the genus demarcation threshold. While the 86% threshold was an approximation and not strictly unique, and species within the same genus might have different evolutionary rates (Ramette and Tiedje, 2007; Liang et al., 2021), therefore, we proposed to tentatively place *R. populi* K-38^T^ in *Allorhizobium* clade 2 and assign the three strains to a novel genus. In addition, the chemotaxonomic and physiological analyses revealed that the three strains shared similar major phenotypic features with each other (Table 1 and Supplementary Table S2), which also confirmed the reclassification.

**Table 1:** Differential characteristics of the strain BDR2-2^T^ and closely related type strains.

**Characteristic**	**1**	**2**	**3**
pH range (optimum)	5.0–9.0 (7.0)	5.0–9.0 (6.5–7.0)	6.0–9.0 (7.5)
Temperature range (optimum; °C)	10–41 (28)	4–37 (28)	25–37 (28)
NaCl tolerance (%, w/v)	0–2	0–0.5	0–3
**Utilization of:**			
D-galactose, L-fucose, D-mannitol, D-arabitol			
Citric acid, D-lactic acid methyl ester	–	+	-
D-salicin, quinic acid, methyl pyruvate, propionic acid, D-aspartic acid	–	+	+
α-D-lactose, glycerol, L-histidine, L-pyroglutamic acid, L-serine, N-acetyl-D-glucosamine	+	–	–
Myo-inositol, D-galacturonic acid, D-gluconic acid, D-glucuronic acid	W	–	–
**Enzyme activities:**			
Alkaline phosphatase, β-galactosidase, esterase lipase (C8), lipase (C14)	+	–	–
Esterase (C4), leucine arylamidase, valine arylamidase, cystine arylamidase, β-galactosidase	+	+	–
Trypsin, α-chymotrypsin	+	–	+
α-galactosidase, α-mannosidase, α-fucosidase	–	+	–
**Acid production from:**			
L-rhamnose, D-melibiose	+	–	–
L-arginine, L-ornithine, D-sorbitol	–	–	+
Predominant polar lipids	PE, PG, PC, DPG, PME	PE, PC, PG, DPG, PME	PE, PG, PC, PME, DPG
G + C content (%)	64.5	61.3	64.9

Strains: 1, BDR2-2^T^; 2, Rhizobium borbori DN316^T^ (data from Zhang G. X. et al., 2011, Rozahon et al., 2014); 3, Rhizobium populi K-38^T^ (data from Rozahon et al., 2014); +, Positive; -, negative; W, weakly positive.

The ANI and dDDH values, which are gold standards for species delineation (Liang et al., 2021), were lower in the three strains than the recommended species boundary cutoff values (Supplementary Table S1), which indicated that the three strains should represent three different species in the family *Rhizobiaceae*.

#### 3.2.2. Chemotaxonomic and physiological analyses

The polar lipid profile of the strain BDR2-2^T^ contained phosphatidylethanolamine (PE), phosphatidylglycerol (PG), phosphatidylcholine (PC), diphosphatidylglycerol (DPG), phosphatidyl monomethyl ethanolamine (PME), three unidentified phospholipids (PLs), and three unidentified lipids (Ls; Supplementary Figure S6), which was similar to the profiles obtained for *A. borbori* DN316^T^ and *R. populi* K-38^T^. The presence of PL and the absence of unidentified amino phospholipids (APLs) could distinguish the strain BDR2-2^T^ from *A. borbori* DN316^T^ and *R. populi* K-38^T^. The strain BDR2-2^T^ could also be distinguished from *A. borbori* DN316^T^ and *R. populi* K-38^T^ based on its physiological and chemotaxonomic features: growth conditions, utilization of carbon sources, and enzyme activities, as shown in Table 1. The strain BDR2-2^T^ exhibited a cellular fatty acid profile mainly consisting of summed feature 8 (comprising C_18:1_ω7*c* and/or C_18:1_ω6*c*, 61.9%), C_19:0_ cyclo ω8*c* (11.0%), C_16:0_ (10.1%), C_16:0_ 3-OH (4.6%), and C_18:0_ (4.7%), which was similar to those of reference strains (Supplementary Table S2). Higher C_18:0_ content of the strain BDR2-2^T^ (4.7%) could clearly separate it from *A. borbori* DN316^T^ (0.7%) and *R. populi* K-38^T^ (1.8%). Along with the phylogenetic analyses, the strain BDR2-2^T^ should represent a species of a novel genus within the family *Rhizobiaceae*, for which the name *Ectorhizobium quercum* gen. nov., sp. nov. was proposed.

## 4. Conclusion

Since the low resolution of 16S rRNA phylogeny on the closely related species was an important cause of the taxonomic issue, we, therefore, constructed two genome-based phylogenetic trees, namely concatenated proteins tree and UBCG tree, to resolve the misclassifications. Genome sequences from 138 of the 181 validly published *Rhizobiaceae* species, 18 not validly published *Rhizobiaceae* species were used to confirm the taxonomic status of species in the family *Rhizobiaceae*, five *Brucellaceae* and five *Caulobacterales* were used as the out-group. Along with the phylogenomic metric analyses of cpAAI, eight novel genera, one novel species, and 22 novel combinations were proposed.

### 4.1. Taxonomic level: new genera

#### 4.1.1. Description of *Allopararhizobium* gen. nov.

*Allopararhizobium* [Al.lo.pa.ra.rhi.zo'bi.um. Gr. masc. adj. *allos*, another, other, different; N.L. neut. n. *Pararhizobium*, a bacterial generic name; N.L. neut. n. *Allopararhizobium*, a genus different from *Pararhizobium*].

Cells are Gram-strain-negative, motile, aerobic, and rod-shaped. The predominant respiratory quinone is Q-10. The major cellular fatty acids usually contain C_19:0_ cyclo ω8*c*. The DNA G+C content is 64.7 mol%. Species of the genus are classified based on UBCG and concatenated protein phylogenetic trees, as well as phylogenomic metric analyses of cpAAI. The type species is *Allopararhizobium mangrovi* comb. nov.

#### 4.1.2. Description of *Paramartelella* gen. nov.

*Paramartelella* [Pa.ra.mar.tel.el'la. Gr. pref. *para-*, beside; N.L. fem. dim. n. *Martelella*, a bacterial generic name; N.L. fem. dim. n. *Paramartelella*, resembling the genus *Martelella*].

Cells are Gram-strain-negative, non-motile, catalase-positive, and rod-shaped. The predominant respiratory quinone is Q-10. The major cellular fatty acids usually contain summed feature 8 (comprising C_18:1_ω7*c* and/or C_18:1_ω6*c*). The DNA G+C content is 62.3 mol%. Species of the genus are classified based on UBCG and concatenated protein phylogenetic trees, as well as phylogenomic metric analyses of cpAAI. The type species is *Paramartelella alba* comb. nov.

#### 4.1.3. Description of *Metarhizobium* gen. nov.

*Metarhizobium* [Me.ta.rhi.zo.bi.um. Gr. adv. *meta*, besides; N.L. neut. n. *Rhizobium*, a bacterial generic name; N.L. neut. n. *Metarhizobium*, a genus besides *Rhizobium*].

Cells are Gram-strain-negative, motile, facultatively anaerobic, catalase- and oxidase-positive, and rod-shaped. The predominant respiratory quinone is Q-10. The major cellular fatty acids usually contain C_19:0_ cyclo ω8*c* and C_18:1_ω7*c*. The DNA G+C content is 61.9 mol%. Species of the genus are classified based on UBCG and concatenated protein phylogenetic trees, as well as phylogenomic metric analyses of cpAAI. The type species is *Metarhizobium album* comb. nov.

#### 4.1.4. Description of *Heterorhizobium* gen. nov.

*Heterorhizobium* [He.te.ro.rhi.zo.bi.um. Gr. masc. adj. *heteros*, different; N.L. neut. n. *Rhizobium*, a bacterial generic name; N.L. neut. n. *Heterorhizobium*, organism different from but related to the genus *Rhizobium*].

Cells are Gram-strain-negative, motile, catalase- and oxidase-positive, aerobic, and rod-shaped. The respiratory quinone is Q-10. The major cellular fatty acids usually contain C_18:1_ω7*c* and C_19:0_ cyclo ω8*c*. The DNA G+C content is 52.8 mol%. Species of the genus are classified based on UBCG and concatenated protein phylogenetic trees, as well as phylogenomic metric analyses of cpAAI. The type species is *Heterorhizobium halophytocola* comb. nov.

#### 4.1.5. Description of *Paenirhizobium* gen. nov.

*Paenirhizobium* [Pae.ni.rhi.zo.bi.um. L. adv. *Paene*, almost; N.L. neut. n. *Rhizobium*, a bacterial generic name; N.L. neut. n. *Paenirhizobium*, almost *Rhizobium*].

Cells are Gram-strain-negative, motile, aerobic, catalase- and oxidase-positive, and rod-shaped. The predominant respiratory quinone is Q-10. The major cellular fatty acids usually contain summed feature 8 (comprising C_18:1_ω7*c* and/or C_18:1_ω6*c*). The DNA G + C content is 60.1–60.9 mol%. Members of the genus are classified based on UBCG and concatenated protein phylogenetic trees, as well as phylogenomic metric analyses of cpAAI. The type species is *Paenirhizobium daejeonense* comb. nov.

#### 4.1.6. Description of *Ectorhizobium* gen. nov.

*Ectorhizobium* [Ec.to.rhi.zo.bi.um. Gr. prep. *ecto*, outside; N.L. neut. n. *Rhizobium*, a bacterial generic name; N.L. neut. n. *Ectorhizobium*, outside of *Rhizobium*].

Cells are Gram-stain-negative, aerobic, catalase-, and oxidase-positive. The predominant respiratory quinone is Q-10. The major cellular fatty acids usually contain summed feature 8 (comprising C_18:1_ω7*c* and/or C_18:1_ω6*c*) and C_16:0._ The DNA G+C content is 61.3–64.5 mol%. Members of the genus are classified based on UBCG and concatenated protein phylogenetic trees, as well as phylogenomic metric analyses of cpAAI. The type species is *Ectorhizobium quercum* sp. nov.

#### 4.1.7. Description of *Affinirhizobium* gen. nov.

*Affinirhizobium* [Af.fi.ni.rhi.zo.bi.um. L. masc./fem. adj. *affinis*, associated with, adjacent; N.L. neut. n. *Rhizobium*, a bacterial generic name; N.L. neut. n. *Affinirhizobium*, a genus associated with *Rhizobium*].

Cells are Gram-strain-negative, catalase-positive, aerobic, and rod-shaped. The DNA G + C content is 59.3–60.2 mol%. The major cellular fatty acids usually contain summed feature 8 (comprising C_18:1_ω7*c* and/or C_18:1_ω6*c*). Species of the genus are classified based on UBCG and concatenated protein phylogenetic trees, as well as phylogenomic metric analyses of cpAAI. The type species is *Affinirhizobium pseudoryzae* comb. nov.

#### 4.1.8. Description of *Alirhizobium* gen. nov.

*Alirhizobium* [A.li.rhi.zo.bi.um. L. masc.pron. *alinus*, other, another; N.L. neut. n. *Rhizobium*, a bacterial generic name; N.L. neut. n. *Alirhizobium*, the other *Rhizobium*].

Cells are Gram-strain-negative, aerobic, positive for oxidase, and rod-shaped. The major cellular fatty acids usually contain summed feature 8 (comprising C_18:1_ω7*c* and/or C_18:1_ω6*c*) and C_16:0_. The DNA G + C content is 58.8–59.0 mol%. Species of the genus are classified based on UBCG and concatenated protein phylogenetic trees, as well as phylogenomic metric analyses of cpAAI. The type species is *Alirhizobium cellulosilyticum* comb. nov.

### 4.2. Taxonomic level: new species

#### 4.2.1. Description of *Ectorhizobium quercum* sp. nov.

*Ectorhizobium quercum* [quer'cum. N.L. gen. neut. n. *quercum*, of oak, of quercus tree].

Cells are Gram-stain-negative, motile with a single polar flagellum, aerobic, catalase-, and oxidase-positive, 0.8–1.2 mm in length, and 0.6–0.7 mm in width. Colonies are milky white, circular, and smooth after incubation for 2 days at 28°C on YMA. The strains grow at 10–41°C (optimum, 28°C), pH 5.0–9.5 (optimum, pH 7), and a concentration of 0–2% (w/v) NaCl. In the API ZYM test, N-acetyl-β-glucosaminidase, β-glucuronidase, α-fucosidase, α-mannosidase, and α-galactosidase are negative, and the rest are positive. In the API 20E, the results are positive for inositol, D-sucrose, sodium pyruvate, D-mannitol, D-glucose, D-melibiose, L-arabinose, and L-rhamnose and negative for the rest. In the API 20NE, the results are positive for D-mannitol, D-glucose, esculin ferric citrate, 4-nitrophenyl-β-D-galactopyranoside, D-mannose, D-maltose, L-arabinose, and malic acid, and negative for the rest. In the Biolog GN2 test, the results are positive for acetic acid, D-cellobiose, L-pyroglutamic acid, α-D-lactose, N-acetyl-D-glucosamine, bromo-succinic acid, α-D-glucose, D-galactose, D-turanose, L-fucose, D-sorbitol, D-arabitol, D-fructose-6-PO4, L-alanine, L-glutamic acid, L-histidine, D-mannitol, lincomycin, pectin, L-galactonic acid lactone, D-mannose, α-keto-glutaric acid, D-fructose, L-malic acid, acetoacetic acid, glycerol, dextrin, L-serine, D-maltose, Tween 40, glucuronamide, weakly positive for D-glucuronic acid, N-acetyl-β-D-mannosamine, N-acetyl-D-galactosamine, D-galacturonic acid, D-fucose, L-arginine, sucrose, D-melibiose, L-aspartic acid, D-gluconic acid, L-lactic acid, D-trehalose, L-rhamnose, myo-inositol, and the rest are negative. The polar lipids are PE, PG, PC, DPG, PME, three unidentified phospholipids (PLs), and three unidentified lipids (L). The respiratory quinones are Q-10. The predominant fatty acids are summed feature 8 (comprising C_18:1_ω7*c* and/or C_18:1_ω6*c*, 61.9%), C_19:0_ cyclo ω8*c* (11.0%), C_16:0_ (10.1%), C_16:0_ 3-OH (4.6%), and C_18:0_ (4.7%). The type strain is BDR2-2^T^ (= CFCC 16492^T^ = LMG 31717^T^), isolated from the symptomatic bark of *Q. acutissima* caker in Anhui province, China. The strain BDR2-2^T^ is predicted to have 4,685 coding genes, three rRNA genes, 54 tRNA genes, and six other RNA genes, and the DNA G+C content is 64.5 mol%.

### 4.3. Taxonomic level: new (combinations for) species

#### 4.3.1. Description of *Allopararhizobium mangrovi* comb.nov.

*Allopararhizobium mangrovi* (man.gro'vi. N.L. gen. neut. n. *mangrovi*, of mangrove, where the bacterium was isolated).

Basonym: *Pararhizobium mangrovi* Li et al., 2021.

The description of *A. mangrovi* is the same as that given for *P. mangrovi* (Li et al., 2021b). The species are classified based on UBCG and concatenated protein phylogenetic trees, as well as phylogenomic metric analyses of cpAAI. The type strain is BGMRC 6574^T^ (= CGMCC 1.16783^T^ = KCTC 72636^T^).

#### 4.3.2. Description of *Paramartelella alba* comb. nov.

*Paramartelella alba* (al'ba. L. fem. adj. *alba*, white, referring to the color of the colonies).

Basonym: *Martelella alba* Li et al., 2021.

The description of *P. alba* is the same as that given for *M. alba* (Li et al., 2021a). The species are classified based on UBCG and concatenated protein phylogenetic trees, as well as phylogenomic metric analyses of cpAAI. The type strain is BGMRC 2036^T^ (= KCTC 52121^T^ = NBRC 111908^T^).

#### 4.3.3. Description of *Mycoplana rhizolycopersici* comb.nov.

*Mycoplana rhizolycopersici* (rhi.zo.ly.co.per'si.ci. Gr. fem. n. *rhiza*, a root; N.L. gen. neut. n. *lycopersici*, of Solanum lycopersicum, the scientific name of the tomato; N.L. gen. neut. n. *rhizolycopersici*, of tomato roots).

Basonym: *Rhizobium rhizolycopersici* Thin et al., 2021.

The description of *M. rhizolycopersici* is the same as that given for *R. rhizolycopersici* (Thin et al., 2021). The species are classified based on UBCG and concatenated protein phylogenetic trees, as well as phylogenomic metric analyses of cpAAI. The type strain is DBTS2^T^ (= CICC 24887^T^ = ACCC61707^T^ = JCM 34245^T^).

#### 4.3.4. Description of *Metarhizobium album* comb. nov.

*Metarhizobium album* (al'bum. L. neut. adj. *album*, white, referring to the white colonies of the organism).

Basonym: *Rhizobium album* Hang et al., 2019.

The description of the *Metarhizobium album* is the same as that given for the *Rhizobium album* (Hang et al., 2019). The species are classified based on UBCG and concatenated protein phylogenetic trees, as well as phylogenomic metric analyses of cpAAI. The type strain is NS-104^T^ (= CCTCC AB 2017250^T^ = KCTC 62327^T^).

#### 4.3.5. Description of *Heterorhizobium halophytocola* comb.nov.

*Heterorhizobium halophytocola* [ha.lo.phy.to'co.la. Gr. masc. n. *hals (gen. halos)*, salt; Gr. neut. n. *phyton*, a plant; L. masc./fem. suff. *-cola*, inhabitant, dweller; from L. masc./fem. n. *incola*, dweller; N.L. masc./fem. n. *halophytocola*, inhabitant of a halophyte, Rosa rugosa (nominative in apposition)].

Basonym: *Rhizobium halophytocola* Bibi et al., 2012.

The description of *H. halophytocola* is the same as that given for *R. halophytocola* (Bibi et al., 2012). The species are classified based on UBCG and concatenated protein phylogenetic trees, as well as phylogenomic metric analyses of cpAAI. The type strain is YC6881^T^ (= DSM 21600^T^ = KACC 13775^T^).

#### 4.3.6. Description of *Paenirhizobium daejeonense* comb.nov.

*Paenirhizobium daejeonense* (dae.jeon.en'se. N.L. neut. adj. *daejeonense*, pertaining to Daejeon, a city in Korea, where the type strain was isolated).

Basonym: *Rhizobium daejeonense* Quan et al., 2005.

The description of *Paenirhizobium daejeonense* is the same as that given for *Rhizobium daejeonense* (Quan et al., 2005). The species are classified based on UBCG and concatenated protein phylogenetic trees, as well as phylogenomic metric analyses of cpAAI. The type strain is L61^T^ (= DSM 17795^T^ = JCM 21505^T^ = IAM 15042^T^ = CCBAU 10050^T^ = NBRC 102495^T^ = KCTC 12121^T^).

#### 4.3.7. Description of *Paenirhizobium naphthalenivorans* comb.nov.

*Paenirhizobium naphthalenivorans* (naph.tha.le.ni.vo'rans. N.L. neut. n. *naphthalenum*, naphthalene; L. pres. part. *vorans*, devouring; N.L. part. adj. *naphthalenivorans*, naphthalene-devouring).

Basonym: *Rhizobium naphthalenivorans* Kaiya et al., 2018.

The description of *Paenirhizobium naphthalenivorans* is the same as that given for *Rhizobium naphthalenivorans* (Kaiya et al., 2012). The species are classified based on UBCG and concatenated protein phylogenetic trees, as well as phylogenomic metric analyses of cpAAI. The type strain is TSY03b^T^ (= KCTC 23252^T^ = NBRC 107585^T^).

#### 4.3.8. Description of *Paenirhizobium selenitireducens* comb.nov.

*Paenirhizobium selenitireducens* (se.le.ni.ti.re.du'cens. N.L. masc. n. *selenis*, selenite; L. pres. part. *reducens*, converting to a different state; N.L. part. adj. *selenitireducens*, selenite reducing, referring to the organism's ability to reduce the selenium oxyanion selenite to elemental selenium).

Basonym: *Rhizobium selenitireducens* Hunter et al., 2008.

The description of *Paenirhizobium selenitireducens* is the same as that given for *Rhizobium selenitireducens* (Hunter et al., 2007). The species are classified based on UBCG and concatenated protein phylogenetic trees, as well as phylogenomic metric analyses of cpAAI. The type strain is B1^T^ (= NRRL B-41997^T^ = LMG 24075^T^ = ATCC BAA-1503^T^).

#### 4.3.9. Description of *Peteryoungia glycinendophyticum* comb.nov.

*Peteryoungia glycinendophyticum* (gly.cin.en.do.phy'ti.cum. N.L. fem. n. *Glycine*, generic name of the soy bean; Gr. pref. *endo-*, within; Gr. neut. n. *phyton*, plant; L. masc. adj. suff. *-icus*, used with the sense of belonging to; N.L. masc. adj. *endophyticus*, within the plant, endophytic; N.L. neut. adj. *glycinendophyticum*, an endophyte of soybean).

Basonym: *Rhizobium glycinendophyticum* Wang et al., 2020.

The description of *P. glycinendophyticum* is the same as that given for *R. glycinendophyticum* (Wang et al., 2020). The species are classified based on UBCG and concatenated protein phylogenetic trees, as well as phylogenomic metric analyses of cpAAI. The type strain is CL12^T^ (= KACC 21281^T^ = GDMCC 1.1597^T^).

#### 4.3.10. Description of *Peteryoungia albertimagni* comb.nov.

*Peteryoungia albertimagni* (*albertimagni*, is named after the Dominican scholar Albertus Magnus, who was the first person to describe arsenic).

Basonym: *Agrobacterium albertimagni* Salmassi et al., 2002.

The description of *P. albertimagni* is the same as that given for *A. albertimagni* (Salmassi et al., 2002). The species are classified based on UBCG and concatenated protein phylogenetic trees, as well as phylogenomic metric analyses of cpAAI. The type strain is AOL15^T^ = ATCC BAA-24^T^).

#### 4.3.11. Description of *Ectorhizobium borbori* comb.nov.

*Ectorhizobium borbori* (bor'bo.ri. Gr. masc. n. *borboros*, sludge; N.L. gen. n. *borbori*, of sludge).

Basonym: *Allorhizobium borbori* Mousavi et al., 2016.

Homotypic synonym: *Rhizobium borbori* Zhang et al., 2011.

The description of *Ectorhizobium borbori* is the same as that given for *Rhizobium borbori* (Zhang G. X. et al., 2011). The species are classified based on UBCG and concatenated protein phylogenetic trees, as well as phylogenomic metric analyses of cpAAI. The type strain is DN316^T^ (= CICC 10378^T^= LMG 23925^T^= DSM 22790^T^=DSM 26385^T^=HAMBI 3454^T^).

#### 4.3.12. Description of *Ectorhizobium populi* comb.nov.

*Ectorhizobium populi* (po'pu.li. L. gen. fem. n. *populi*, of a poplar tree, pertaining to Populus euphratica, the Latin name for the poplars that grow in the forest from which the type strain was isolated).

Basonym: *Rhizobium populi* Rozahon et al., 2014.

The description of *E. populi* is the same as that given for *R. populi* (Rozahon et al., 2014). The species are classified based on UBCG and concatenated protein phylogenetic trees, as well as phylogenomic metric analyses of cpAAI. The type strain is K-38^T^ (= CCTCC AB 2013068^T^ = NRRL B-59990^T^ = JCM 19159^T^).

#### 4.3.13. Description of *Agrobacterium oryzihabitans* comb.nov.

*Agrobacterium oryzihabitans* (o.ry.zi.ha'bi.tans. L. fem. n. *oryza*, rice; L. pres. part. *habitans*, inhabiting, dwelling; N.L. part. adj. *oryzihabitans*, rice-inhabiting).

Basonym: *Rhizobium oryzihabitans* Zhao et al., 2020.

The description of *A. oryzihabitans* is the same as that given for *R. oryzihabitans* (Zhao et al., 2020). The species are classified based on UBCG and concatenated protein phylogenetic trees, as well as phylogenomic metric analyses of cpAAI. The type strain is M15^T^ (= JCM 32903^T^ = ACCC 60121^T^).

#### 4.3.14. Description of *Affinirhizobium pseudoryzae* comb.nov.

*Affinirhizobium pseudoryzae* (a.qua'ti.cum. L. neut. adj. *aquaticum*, living in water, aquatic, referring to the isolation source of the type strain).

Basonym: *Allorhizobium pseudoryzae* Mousavi et al., 2016.

Homotypic synonym: *Rhizobium pseudoryzae* Zhang G. X. et al., 2011.

The description of *Affinirhizobium pseudoryzae* is the same as that given for *Rhizobium pseudoryzae* (Zhang X. et al., 2011). The species are classified based on UBCG and concatenated protein phylogenetic trees, as well as phylogenomic metric analyses of cpAAI. The type strain is J3-A127^T^ (= ACCC 10380^T^ = KCTC 23294^T^ = DSM 19479^T^ = DSM 26483^T^).

#### 4.3.15. Description of *Affinirhizobium helianthi* comb.nov.

*Affinirhizobium helianthi* (he.li.an'thi. N.L. gen. masc. n. *helianthi*, of the sunflower Helianthus).

Basonym: *Rhizobium helianthi* Wei et al., 2015.

The description of *Affinirhizobium helianthi* is the same as that given for *Rhizobium helianthi* (Wei et al., 2015). The species are classified based on UBCG and concatenated protein phylogenetic trees, as well as phylogenomic metric analyses of cpAAI. The type strain is Xi19^T^ (= CGMCC 1.12192^T^ = KCTC 23879^T^).

#### 4.3.16. Description of *Affinirhizobium rhizoryzae* comb.nov.

*Affinirhizobium rhizoryzae* (rhiz.o.ry'zae. Gr. fem. n. *rhiza*, root; L. gen. fem. n. *oryzae*, of rice; N.L. gen. n. *rhizoryzae*, of rice roots).

Basonym: *Rhizobium rhizoryzae* Zhang et al., 2014.

The description of *Affinirhizobium rhizoryzae* is the same as that given for *Rhizobium rhizoryzae* (Zhang X. X. et al., 2014). The species are classified based on UBCG and concatenated protein phylogenetic trees, as well as phylogenomic metric analyses of cpAAI. The type strain is J3-AN59^T^ (= ACCC 05916^T^ = DSM 19478^T^ = DSM 29514^T^ = KCTC 23652^T^).

#### 4.3.17. Description of *Alirhizobium cellulosilyticum* comb.nov.

*Alirhizobium cellulosilyticum* (cel.lu.lo.si.ly'ti.cum. N.L. neut. N. *cellulosum*, cellulose; N.L. neut. Adj. *lyticum*, dissolving; from Gr. Masc. adj. *lytikos*, able to loose, able to dissolve; N.L. neut. Adj. *cellulosilyticum*, cellulose-dissolving).

Basonym: *Rhizobium cellulosilyticum* Garcia-Fraile et al., 2007.

The description of *Alirhizobium cellulosilyticum* is the same as that given for *Rhizobium cellulosilyticum* (Garcia-Fraile et al., 2007). The species are classified based on UBCG and concatenated protein phylogenetic trees, as well as phylogenomic metric analyses of cpAAI. The type strain is ALA10B2^T^ (=DSM 18291^T^ = CECT 7176^T^ = LMG 23642^T^).

#### 4.3.18. Description of *Alirhizobium wenxiniae* comb.nov.

*Alirhizobium wenxiniae* (wen.xin'i.ae. N.L. gen. fem. n. *wenxiniae*, of Wen-xin, to honor Wen-xin Chen, a respected rhizobial taxonomist, for her great contributions to the investigation and taxonomy of rhizobial resources in China).

Basonym: *Rhizobium wenxiniae* Gao et al., 2017.

The description of *Alirhizobium wenxiniae* is the same as that given for *Rhizobium wenxiniae* (Gao et al., 2017). The species are classified based on UBCG and concatenated protein phylogenetic trees, as well as phylogenomic metric analyses of cpAAI. The type strain is 166^T^ (= DSM 100734^T^ = CGMCC 1.15279^T^).

#### 4.3.19. Description of *Alirhizobium smilacinae* comb.nov.

*Alirhizobium smilacinae* (smi.la.ci'na.e. N.L. fem. n. *Smilacina*, a botanical genus name; N.L. gen. fem. n. *smilacinae*, of the plant genus Smilacina).

Basonym: *Rhizobium smilacinae* Zhang et al., 2015.

The description of *Alirhizobium smilacinae* is the same as that given for *Rhizobium smilacinae* (Zhang L. et al., 2014). The species are classified based on UBCG and concatenated protein phylogenetic trees, as well as phylogenomic metric analyses of cpAAI. The type strain is PTYR-5^T^ (= DSM 100675^T^ = CCTCC AB 2013016^T^ = KCTC 32300^T^ = LMG 27604^T^).

#### 4.3.20. Description of *Neorhizobium deserti* comb. nov.

*Neorhizobium deserti* (de.ser'ti. L. gen. neut. n. *deserti*, of a desert, the source of the type strain).

Basonym: *Rhizobium deserti* Liu et al., 2020.

The description of *N. deserti* is the same as that given for *R. deserti* (Liu et al., 2020). The species are classified based on UBCG and concatenated protein phylogenetic trees, as well as phylogenomic metric analyses of cpAAI. The type strain is SPY-1^T^ (= ACCC 61627^T^ = JCM 33732^T^).

#### 4.3.21. Description of *Neorhizobium terrae* comb. nov.

*Neorhizobium terrae* (ter'rae. L. gen. fem. n. *terrae*, of soil, referring to the isolation source of the type strain).

Basonym: *Rhizobium terrae* Ruan et al., 2021.

The description of *N. terrae* is the same as that given for *R. terrae* (Ruan et al., 2020). The species are classified based on UBCG and concatenated protein phylogenetic trees, as well as phylogenomic metric analyses of cpAAI. The type strain is NAU-18^T^ (= CCTCC AB 2018075^T^ = KCTC 62418^T^).

#### 4.3.22. Description of *Neorhizobium populisoli* comb.nov.

*Neorhizobium populisoli* (po.pu.li.so'li. L. fem. n. *Populus*, the poplar tree (genus Populus); L. neut. adj. *solum*, soil; N.L. gen. neut. n. *populisoli*, of poplar soil, referring to the isolation of the bacterium from the rhizosphere soil of *P. popularis*).

Basonym: *Rhizobium populisoli* Shen et al., 2022.

The description of *N. populisoli* is the same as that given for *R. populisoli* (Shen et al., 2022). The species are classified based on UBCG and concatenated protein phylogenetic trees, as well as phylogenomic metric analyses of cpAAI. The type strain is XQZ8^T^ (=JCM 34442^T^ = GDMCC 1.2201^T^).

## Data availability statement

The datasets presented in this study can be found in online repositories. The names of the repository/repositories and accession number(s) can be found at: NCBI—JANFPI010000000 and PRJNA859997.

## Author contributions

CP and YL designed the experiment, provided the methods, and revised the manuscript. TM finished the manuscript and completed most of the experiments. HX and NJ helped to reconstruct and analyze the gene trees. All authors read and approved the final version of the manuscript.
