# Supplementary material for: Phylogenomic reappraisal of the family Rhizobiaceae at the genus and species levels, including the description of Ectorhizobium quercum gen. nov., sp. nov
Source: Front Microbiol. 2023 Aug 3;14:1207256. doi: 10.3389/fmicb.2023.1207256 (PMC10434624; doi:10.3389/fmicb.2023.1207256)
Supplement: Supplementary file 1 [file Data_Sheet_1.PDF]

Genome-based phylogenetic reappraisal of the family *Rhizobiaceae* at genus and species levels and description of *Ectorhizobium quercum* gen. nov., sp. nov.

Supplementary Figures and Tables

1. 16S rRNA gene phylogenetic tree (Figure S1) 6
2. Concatenated proteins phylogenetic tree (Figure S2) 9
3. UBCG phylogenetic tree (Figure S3) 12
4. The rank order of pairwise cpAAI values of members within family *Rhizobiaceae* (Figure S4-5) 13
5. Polar lipid profiles of the novel strains (Figure S6).. 15
6. Differential characteristics of the novel strain and closely related type strains (Table S2)...16
7. Cellular fatty acid profiles of the novel strain and closely related type strains (TableS3).....17

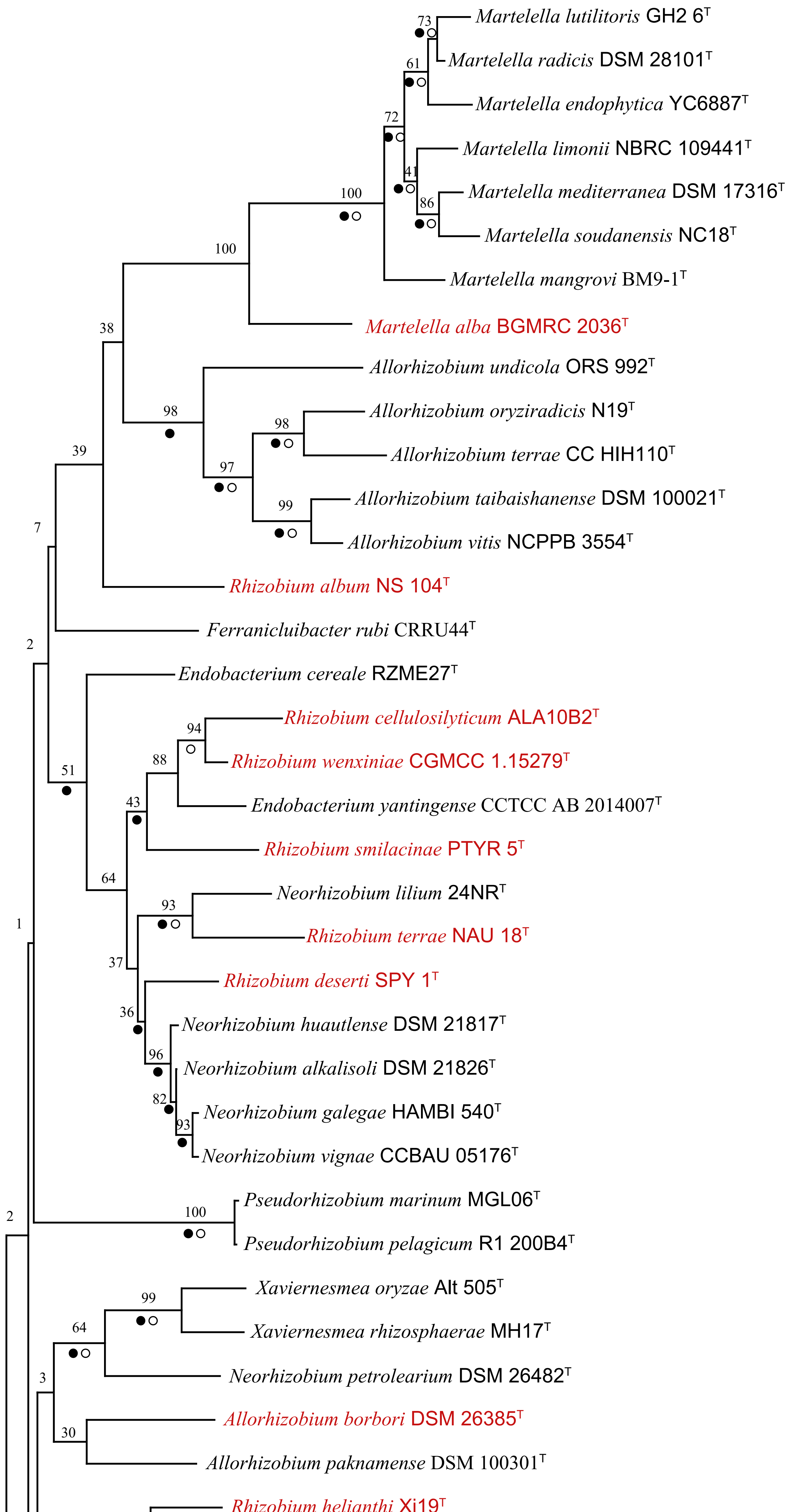
Martelella lutilitoris GH2 6T
Martelella radicis DSM 28101T
Martelella endophytica YC6887T
Martelella limonii NBRC 109441T
Martelella mediterranea DSM 17316T
Martelella soudanensis NC18T
Martelella mangrovi BM9-1T
Martelella alba BGMRC 2036T
Allorhizobium undicola ORS 992T
Allorhizobium oryziradicis N19T
Allorhizobium terrae CC HIH110T
Allorhizobium taibaishanense DSM 100021T
Allorhizobium vitis NCPPB 3554T
Rhizobium album NS 104T
Ferranicluibacter rubi CRRU44T
Endobacterium cereale RZME27T
Rhizobium cellulosilyticum ALA10B2T
Rhizobium wenxiniae CGMCC 1.15279T
Endobacterium yantingense CCTCC AB 2014007T
Rhizobium smilacinae PTYR 5T
Neorhizobium lilium 24NRT
Rhizobium terrae NAU 18T
Rhizobium deserti SPY 1T
Neorhizobium huautlense DSM 21817T
Neorhizobium alkalisoli DSM 21826T
Neorhizobium galegae HAMBI 540T
Neorhizobium vignae CCBAU 05176T
Pseudorhizobium marinum MGL06T
Pseudorhizobium pelagicum R1 200B4T
Xaviernesmea oryzae Alt 505T
Xaviernesmea rhizosphaerae MH17T
Neorhizobium petrolearium DSM 26482T
Allorhizobium borbori DSM 26385T
Allorhizobium paknamense DSM 100301T
Rhizobium helianthi Xi19T

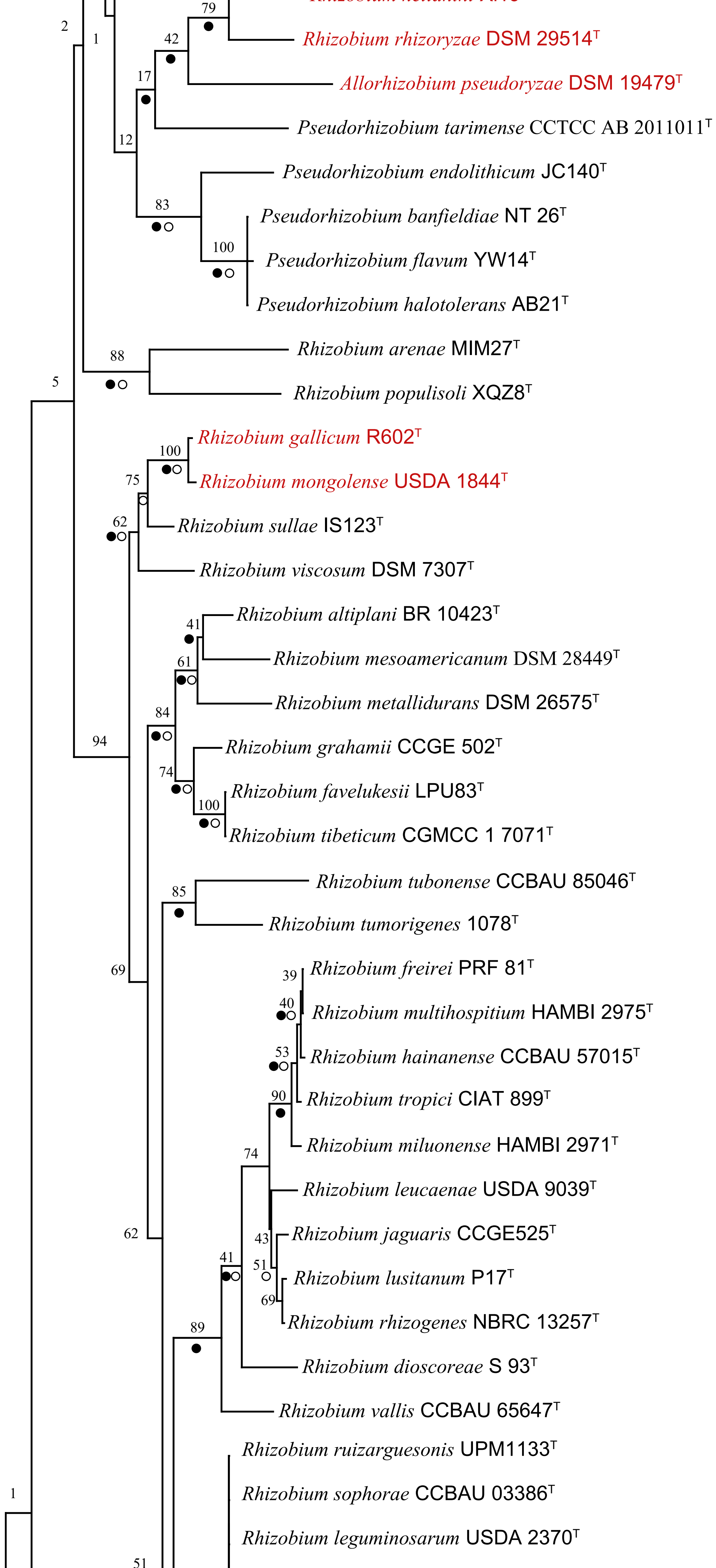

Rhizobium rhizoryzae DSM 29514T
Allorhizobium pseudoryzae DSM 19479T
Pseudorhizobium tarimense CCTCC AB 2011011T
Pseudorhizobium endolithicum JC140T
Pseudorhizobium banfieldiae NT 26T
Pseudorhizobium flavum YW14T
Pseudorhizobium halotolerans AB21T
Rhizobium arenae MIM27T
Rhizobium populisoli XQZ8T
Rhizobium gallicum R602T
Rhizobium mongolense USDA 1844T
Rhizobium sullae IS123T
Rhizobium viscosum DSM 7307T
Rhizobium altiplani BR 10423T
Rhizobium mesoamericanum DSM 28449T
Rhizobium metallidurans DSM 26575T
Rhizobium grahamii CCGE 502T
Rhizobium favelukesii LPU83T
Rhizobium tibeticum CGMCC 1 7071T
Rhizobium tubonense CCBAU 85046T
Rhizobium tumorigenes 1078T
Rhizobium freirei PRF 81T
Rhizobium multihospitium HAMBI 2975T
Rhizobium hainanense CCBAU 57015T
Rhizobium tropici CIAT 899T
Rhizobium miluonense HAMBI 2971T
Rhizobium leucaenae USDA 9039T
Rhizobium jaguaris CCGE525T
Rhizobium lusitanum P17T
Rhizobium rhizogenes NBRC 13257T
Rhizobium dioscoreae S 93T
Rhizobium vallis CCBAU 65647T
Rhizobium ruizarguesonis UPM1133T
Rhizobium sophorae CCBAU 03386T
Rhizobium leguminosarum USDA 2370T
79
2
1
42
17
12
83
100
88
5
100
75
62
41
61
84
94
74
100
85
69
39
40
53
90
74
43
62
41
51
69
89
1
51

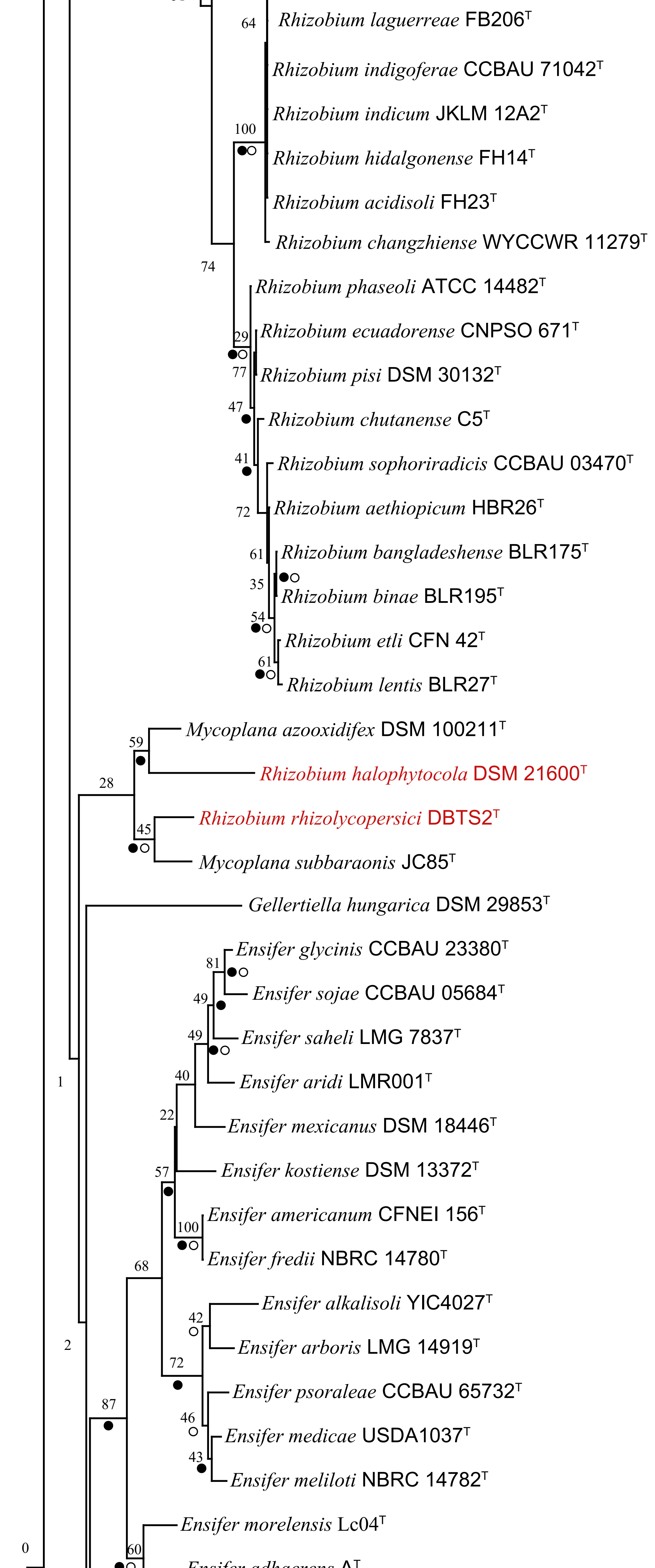
Rhizobium laguerreae FB206T
Rhizobium indigoferae CCBAU 71042T
Rhizobium indicum JKLM 12A2T
Rhizobium hidalgonense FH14T
Rhizobium acidisoli FH23T
Rhizobium changzhiense WYCCWR 11279T
Rhizobium phaseoli ATCC 14482T
Rhizobium ecuadorense CNPSO 671T
Rhizobium pisi DSM 30132T
Rhizobium chutanense C5T
Rhizobium sophoriradicis CCBAU 03470T
Rhizobium aethiopicum HBR26T
Rhizobium bangladeshense BLR175T
Rhizobium binae BLR195T
Rhizobium etli CFN 42T
Rhizobium lentis BLR27T
Mycoplana azooxidifex DSM 100211T
Rhizobium halophytocola DSM 21600T
Rhizobium rhizolycopersici DBTS2T
Mycoplana subbaraonis JC85T
Gellertiella hungarica DSM 29853T
Ensifer glycinis CCBAU 23380T
Ensifer sojae CCBAU 05684T
Ensifer saheli LMG 7837T
Ensifer aridi LMR001T
Ensifer mexicanus DSM 18446T
Ensifer kostiense DSM 13372T
Ensifer americanum CFNEI 156T
Ensifer fredii NBRC 14780T
Ensifer alkalisoli YIC4027T
Ensifer arboris LMG 14919T
Ensifer psoraleae CCBAU 65732T
Ensifer medicae USDA1037T
Ensifer meliloti NBRC 14782T
Ensifer morelensis Lc04T

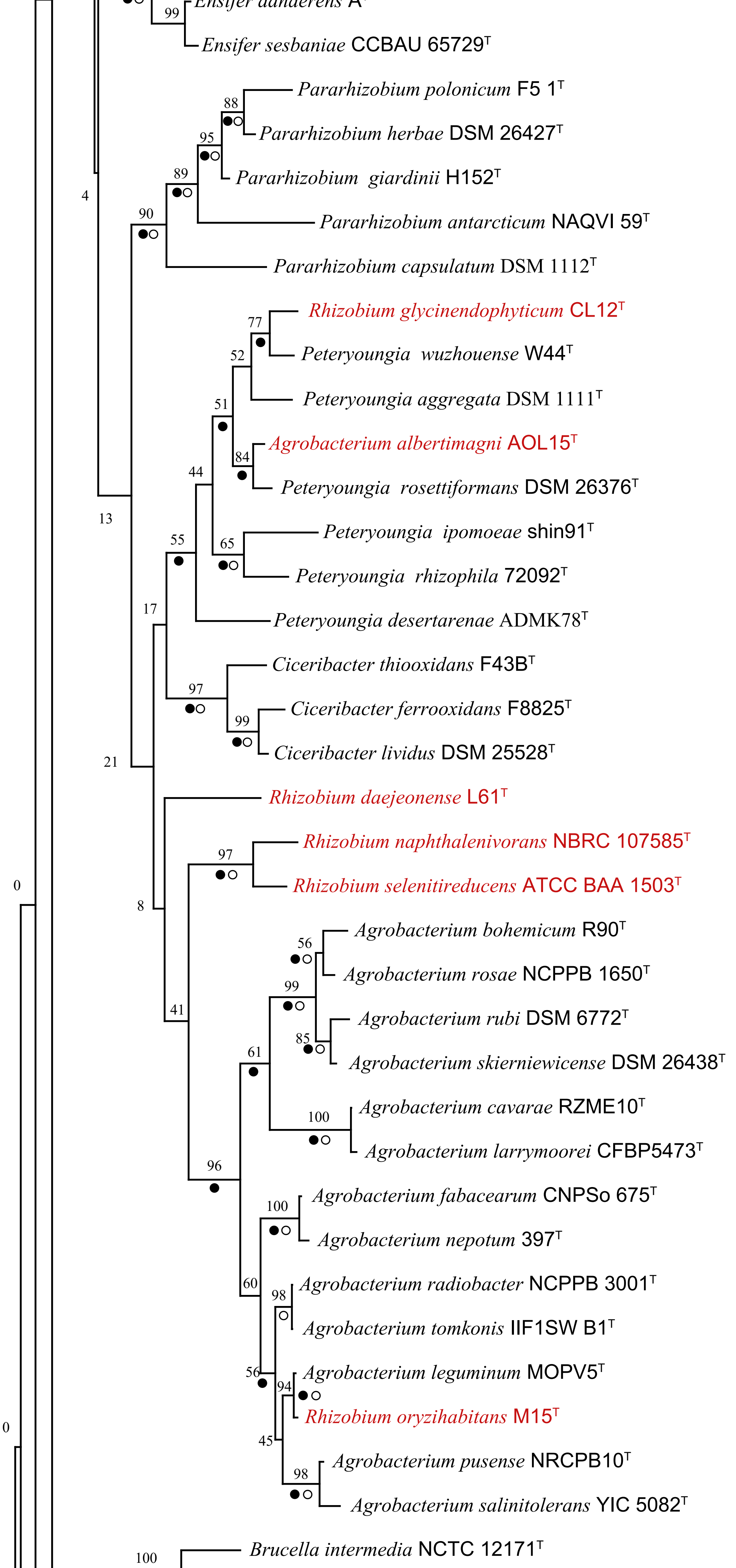
Ensifer sesbaniae CCBAU 65729T
Pararhizobium polonicum F5 1T
Pararhizobium herbae DSM 26427T
Pararhizobium giardinii H152T
Pararhizobium antarcticum NAQVI 59T
Pararhizobium capsulatum DSM 1112T
Rhizobium glycinendophyticum CL12T
Peteryoungia wuzhouense W44T
Peteryoungia aggregata DSM 1111T
Agrobacterium albertimagni AOL15T
Peteryoungia rosettiformans DSM 26376T
Peteryoungia ipomoeae shin91T
Peteryoungia rhizophila 72092T
Peteryoungia desertarenae ADMK78T
Ciceribacter thiooxidans F43BT
Ciceribacter ferrooxidans F8825T
Ciceribacter lividus DSM 25528T
Rhizobium daejeonense L61T
Rhizobium naphthalenivorans NBRC 107585T
Rhizobium selenitireducens ATCC BAA 1503T
Agrobacterium bohemicum R90T
Agrobacterium rosae NCPPB 1650T
Agrobacterium rubi DSM 6772T
Agrobacterium skierniewicense DSM 26438T
Agrobacterium cavarae RZME10T
Agrobacterium larrymoorei CFBP5473T
Agrobacterium fabacearum CNPSo 675T
Agrobacterium nepotum 397T
Agrobacterium radiobacter NCPPB 3001T
Agrobacterium tomkonis IIF1SW B1T
Agrobacterium leguminum MOPV5T
Rhizobium oryzihabitans M15T
Agrobacterium pusense NRCPB10T
Agrobacterium salinitolerans YIC 5082T
Brucella intermedia NCTC 12171T

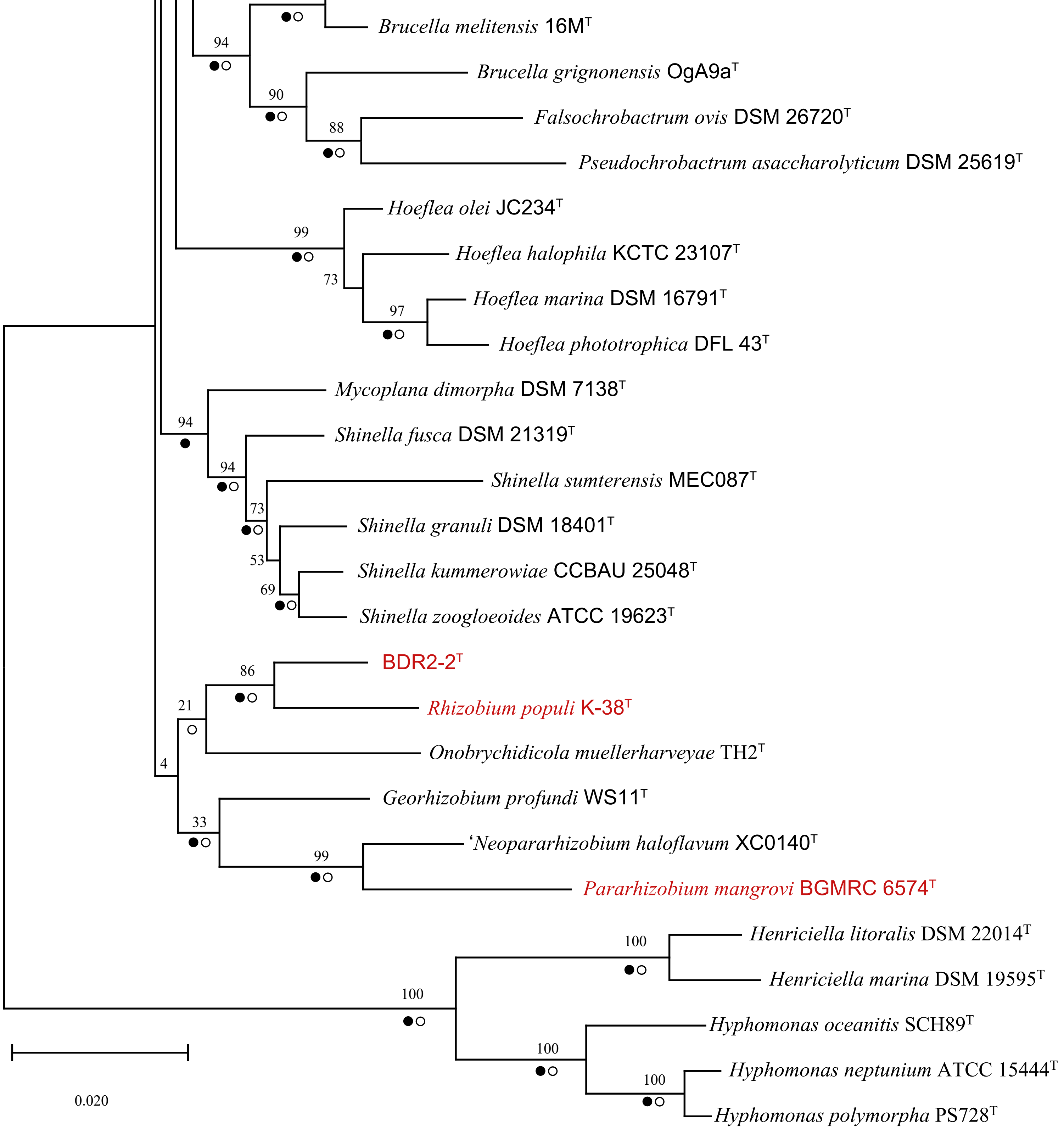

Figure S1. Neighbor-joining tree showing phylogenetic relationships among the strains in family *Rhizobiaceae* based on 16S rRNA gene sequences. The strains of *Brucellaceae* and *Caulobacterales* were used as outgroup. The scale bar corresponds to 0.01 substitutions per nucleotide site. Filled circles indicate branches recovered by maximum-likelihood method and open circles at branches recovered by the maximum parsimony method.

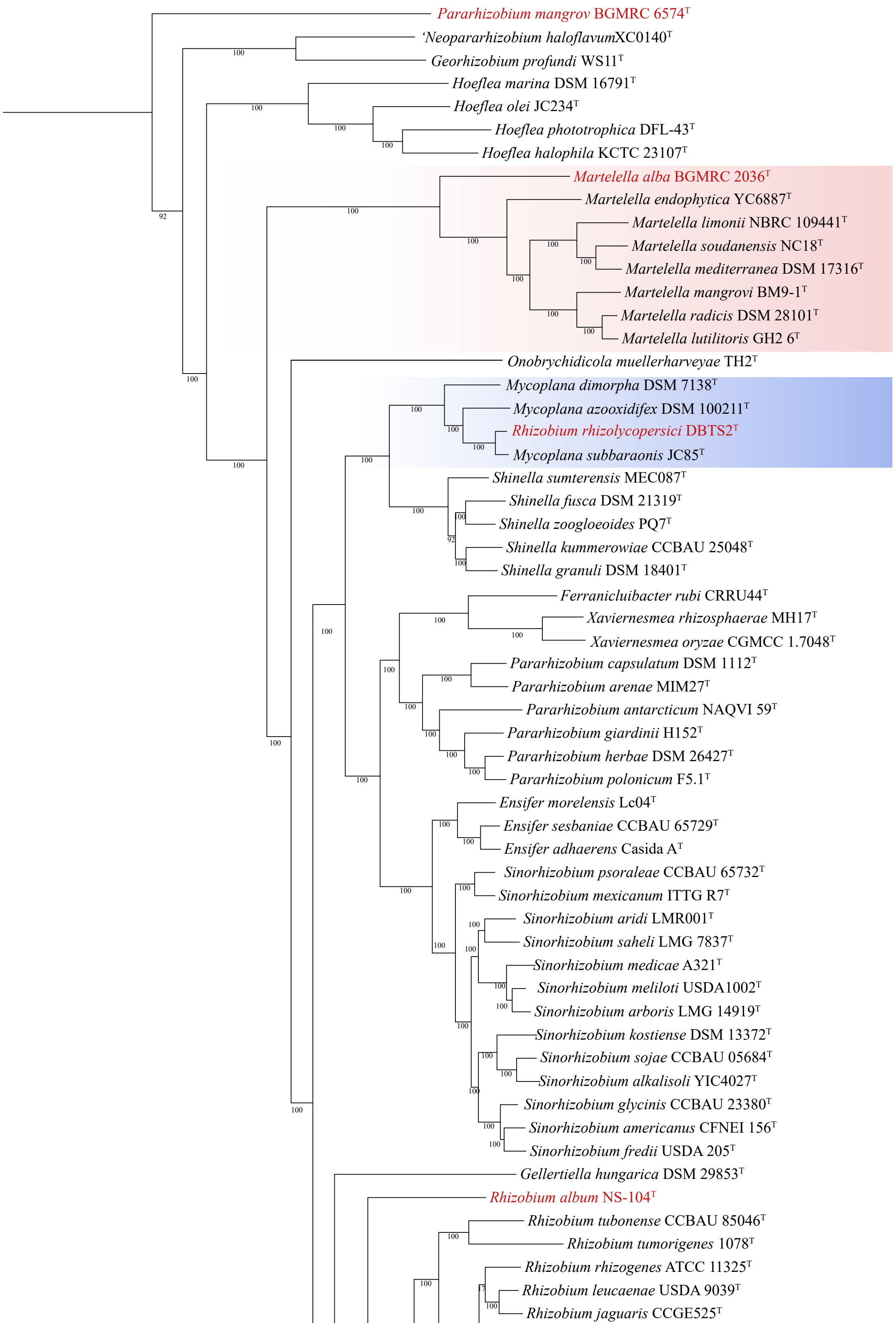
Pararhizobium mangrov BGMRC 6574^{T}
*'Neopararhizobium haloflavum*XC0140^{T}
Georhizobium profundi WS11^{T}
Hoeflea marina DSM 16791^{T}
Hoeflea olei JC234^{T}
Hoeflea phototrophica DFL-43^{T}
Hoeflea halophila KCTC 23107^{T}
Martelella alba BGMRC 2036^{T}
Martelella endophytica YC6887^{T}
Martelella limonii NBRC 109441^{T}
Martelella soudanensis NC18^{T}
Martelella mediterranea DSM 17316^{T}
Martelella mangrovi BM9-1^{T}
Martelella radicis DSM 28101^{T}
Martelella lutilitoris GH2 6^{T}
Onobrychidicola muellerharveyae TH2^{T}
Mycoplana dimorpha DSM 7138^{T}
Mycoplana azooxidifex DSM 100211^{T}
Rhizobium rhizolycopersici DBTS2^{T}
Mycoplana subbaraonis JC85^{T}
Shinella sumterensis MEC087^{T}
Shinella fusca DSM 21319^{T}
Shinella zoogloeoides PQ7^{T}
Shinella kummerowiae CCBAU 25048^{T}
Shinella granuli DSM 18401^{T}
Ferranicluibacter rubi CRRU44^{T}
Xaviernesmea rhizosphaerae MH17^{T}
Xaviernesmea oryzae CGMCC 1.7048^{T}
Pararhizobium capsulatum DSM 1112^{T}
Pararhizobium arenae MIM27^{T}
Pararhizobium antarcticum NAQVI 59^{T}
Pararhizobium giardinii H152^{T}
Pararhizobium herbae DSM 26427^{T}
Pararhizobium polonicum F5.1^{T}
Ensifer morelensis Lc04^{T}
Ensifer sesbaniae CCBAU 65729^{T}
Ensifer adhaerens Casida A^{T}
Sinorhizobium psoraleae CCBAU 65732^{T}
Sinorhizobium mexicanum ITTG R7^{T}
Sinorhizobium aridi LMR001^{T}
Sinorhizobium saheli LMG 7837^{T}
Sinorhizobium medicae A321^{T}
Sinorhizobium meliloti USDA1002^{T}
Sinorhizobium arboris LMG 14919^{T}
Sinorhizobium kostiense DSM 13372^{T}
Sinorhizobium sojae CCBAU 05684^{T}
Sinorhizobium alkalisoli YIC4027^{T}
Sinorhizobium glycinis CCBAU 23380^{T}
Sinorhizobium americanus CFNEI 156^{T}
Sinorhizobium fredii USDA 205^{T}
Gellertiella hungarica DSM 29853^{T}
Rhizobium album NS-104^{T}
Rhizobium tubonense CCBAU 85046^{T}
Rhizobium tumorigenes 1078^{T}
Rhizobium rhizogenes ATCC 11325^{T}
Rhizobium leucaenae USDA 9039^{T}
Rhizobium jaguaris CCGE525^{T}

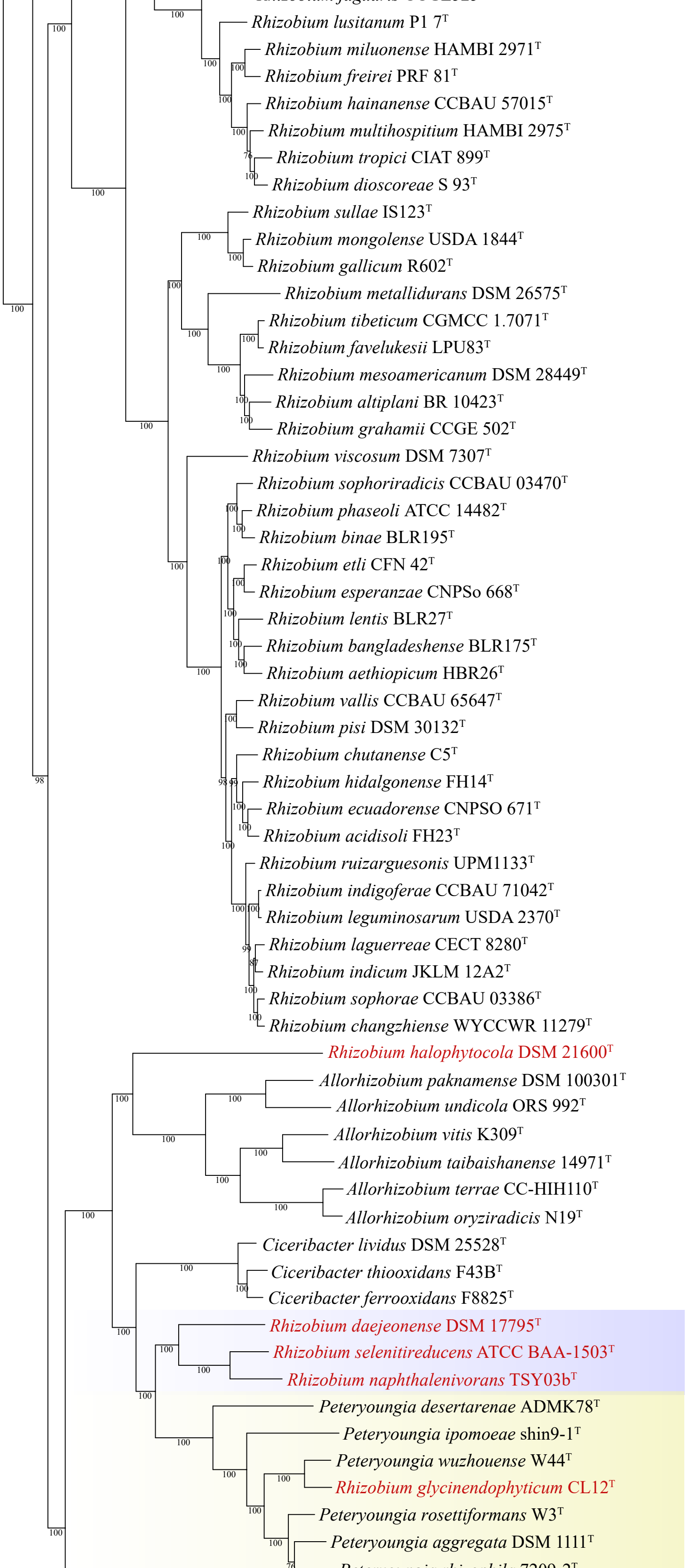
Rhizobium lusitanum P1 7T
Rhizobium miluonense HAMBI 2971T
Rhizobium freirei PRF 81T
Rhizobium hainanense CCBAU 57015T
Rhizobium multihospitium HAMBI 2975T
Rhizobium tropici CIAT 899T
Rhizobium dioscoreae S 93T
Rhizobium sullae IS123T
Rhizobium mongolense USDA 1844T
Rhizobium gallicum R602T
Rhizobium metallidurans DSM 26575T
Rhizobium tibeticum CGMCC 1.7071T
Rhizobium favelukesii LPU83T
Rhizobium mesoamericanum DSM 28449T
Rhizobium altiplani BR 10423T
Rhizobium grahamii CCGE 502T
Rhizobium viscosum DSM 7307T
Rhizobium sophoriradicis CCBAU 03470T
Rhizobium phaseoli ATCC 14482T
Rhizobium binae BLR195T
Rhizobium etli CFN 42T
Rhizobium esperanzae CNPSo 668T
Rhizobium lentis BLR27T
Rhizobium bangladeshense BLR175T
Rhizobium aethiopicum HBR26T
Rhizobium vallis CCBAU 65647T
Rhizobium pisi DSM 30132T
Rhizobium chutanense C5T
Rhizobium hidalgonense FH14T
Rhizobium ecuadorense CNPSO 671T
Rhizobium acidisoli FH23T
Rhizobium ruizarguesonis UPM1133T
Rhizobium indigoferae CCBAU 71042T
Rhizobium leguminosarum USDA 2370T
Rhizobium laguerreae CECT 8280T
Rhizobium indicum JKLM 12A2T
Rhizobium sophorae CCBAU 03386T
Rhizobium changzhiense WYCCWR 11279T
Rhizobium halophytocola DSM 21600T
Allorhizobium paknamense DSM 100301T
Allorhizobium undicola ORS 992T
Allorhizobium vitis K309T
Allorhizobium taibaishanense 14971T
Allorhizobium terrae CC-HIH110T
Allorhizobium oryziradicis N19T
Ciceribacter lividus DSM 25528T
Ciceribacter thiooxidans F43BT
Ciceribacter ferrooxidans F8825T
Rhizobium daejeonense DSM 17795T
Rhizobium selenitireducens ATCC BAA-1503T
Rhizobium naphthalenivorans TSY03bT
Peteryoungia desertarenae ADMK78T
Peteryoungia ipomoeae shin9-1T
Peteryoungia wuzhouense W44T
Rhizobium glycinendophyticum CL12T
Peteryoungia rosettiformans W3T
Peteryoungia aggregata DSM 1111T

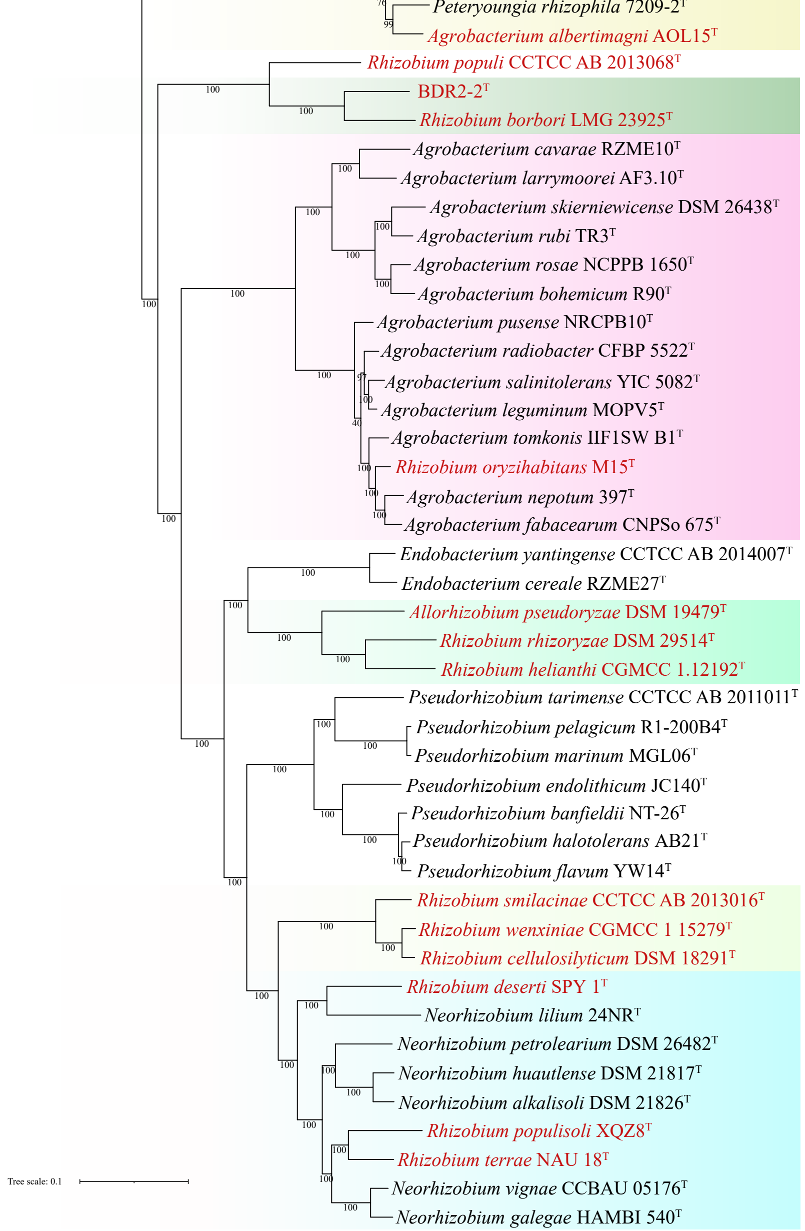

Figure S2. The concatenated proteins phylogenetic tree among strains in family *Rhizobiaceae* based on a concatenated alignment of 170 single-copy proteins. The scale bar corresponds to 0.1 substitutions per amino acid position.

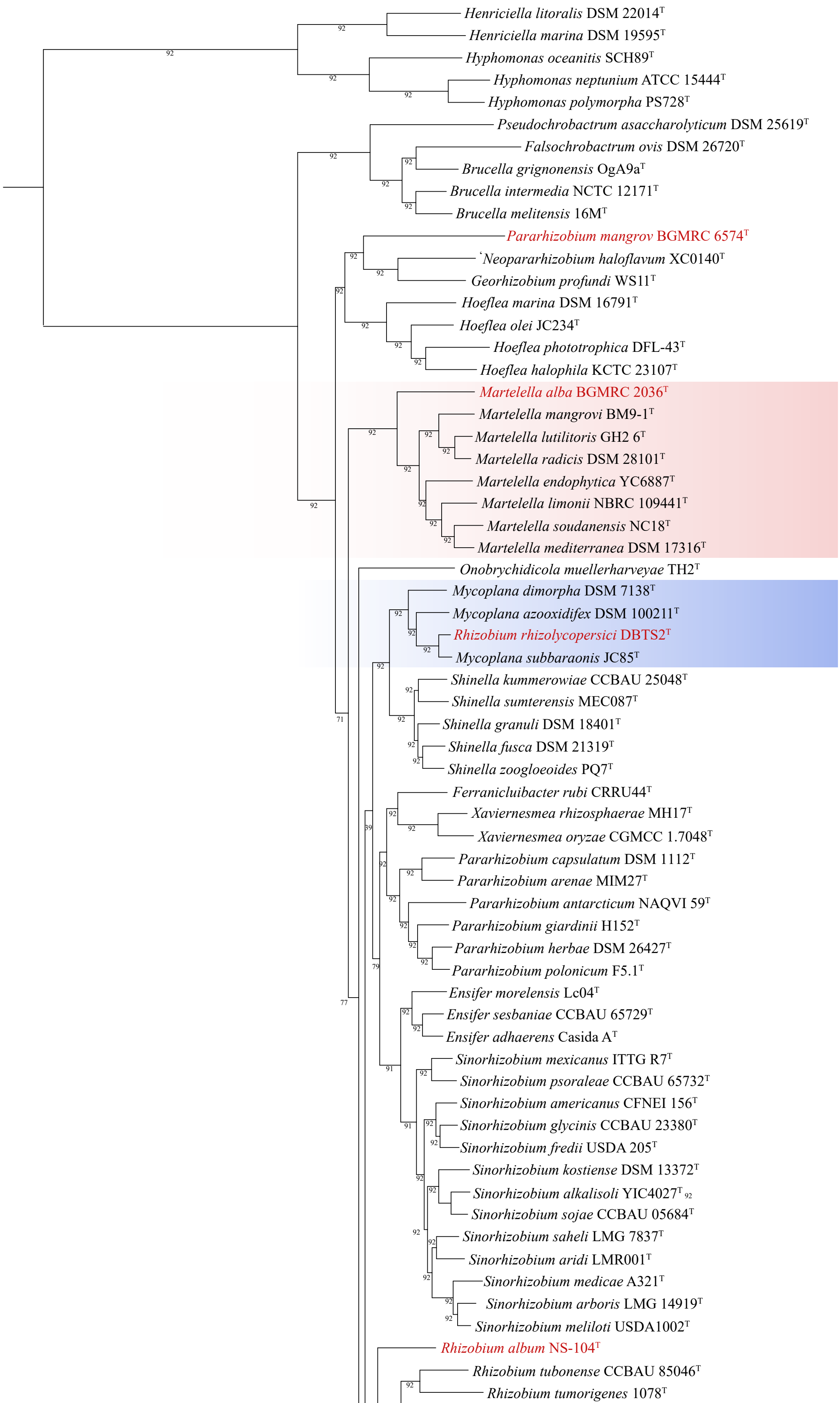

Henriciella litoralis DSM 22014^{T}
Henriciella marina DSM 19595^{T}
Hyphomonas oceanitis SCH89^{T}
Hyphomonas neptunium ATCC 15444^{T}
Hyphomonas polymorpha PS728^{T}
Pseudochrobactrum asaccharolyticum DSM 25619^{T}
Falsochrobactrum ovis DSM 26720^{T}
Brucella grignonensis OgA9a^{T}
Brucella intermedia NCTC 12171^{T}
Brucella melitensis 16M^{T}
Pararhizobium mangrov BGMRC 6574^{T}
Neopararhizobium haloflavum XC0140^{T}
Georhizobium profundi WS11^{T}
Hoeflea marina DSM 16791^{T}
Hoeflea olei JC234^{T}
Hoeflea phototrophica DFL-43^{T}
Hoeflea halophila KCTC 23107^{T}
Martelella alba BGMRC 2036^{T}
Martelella mangrovi BM9-1^{T}
Martelella lutilitoris GH2 6^{T}
Martelella radicis DSM 28101^{T}
Martelella endophytica YC6887^{T}
Martelella limonii NBRC 109441^{T}
Martelella soudanensis NC18^{T}
Martelella mediterranea DSM 17316^{T}
Onobrychidicola muellerharveyae TH2^{T}
Mycoplana dimorpha DSM 7138^{T}
Mycoplana azooxidifex DSM 100211^{T}
Rhizobium rhizolycopersici DBTS2^{T}
Mycoplana subbaraonis JC85^{T}
Shinella kummerowiae CCBAU 25048^{T}
Shinella sumterensis MEC087^{T}
Shinella granuli DSM 18401^{T}
Shinella fusca DSM 21319^{T}
Shinella zoogloeoides PQ7^{T}
Ferranicluibacter rubi CRRU44^{T}
Xaviernesmea rhizosphaerae MH17^{T}
Xaviernesmea oryzae CGMCC 1.7048^{T}
Pararhizobium capsulatum DSM 1112^{T}
Pararhizobium arenae MIM27^{T}
Pararhizobium antarcticum NAQVI 59^{T}
Pararhizobium giardinii H152^{T}
Pararhizobium herbae DSM 26427^{T}
Pararhizobium polonicum F5.1^{T}
Ensifer morelensis Lc04^{T}
Ensifer sesbaniae CCBAU 65729^{T}
Ensifer adhaerens Casida A^{T}
Sinorhizobium mexicanus ITTG R7^{T}
Sinorhizobium psoraleae CCBAU 65732^{T}
Sinorhizobium americanus CFNEI 156^{T}
Sinorhizobium glycinis CCBAU 23380^{T}
Sinorhizobium fredii USDA 205^{T}
Sinorhizobium kostiense DSM 13372^{T}
Sinorhizobium alkalisoli YIC4027^{T}
Sinorhizobium sojae CCBAU 05684^{T}
Sinorhizobium saheli LMG 7837^{T}
Sinorhizobium aridi LMR001^{T}
Sinorhizobium medicae A321^{T}
Sinorhizobium arboris LMG 14919^{T}
Sinorhizobium meliloti USDA1002^{T}
Rhizobium album NS-104^{T}
Rhizobium tubonense CCBAU 85046^{T}
Rhizobium tumorigenes 1078^{T}

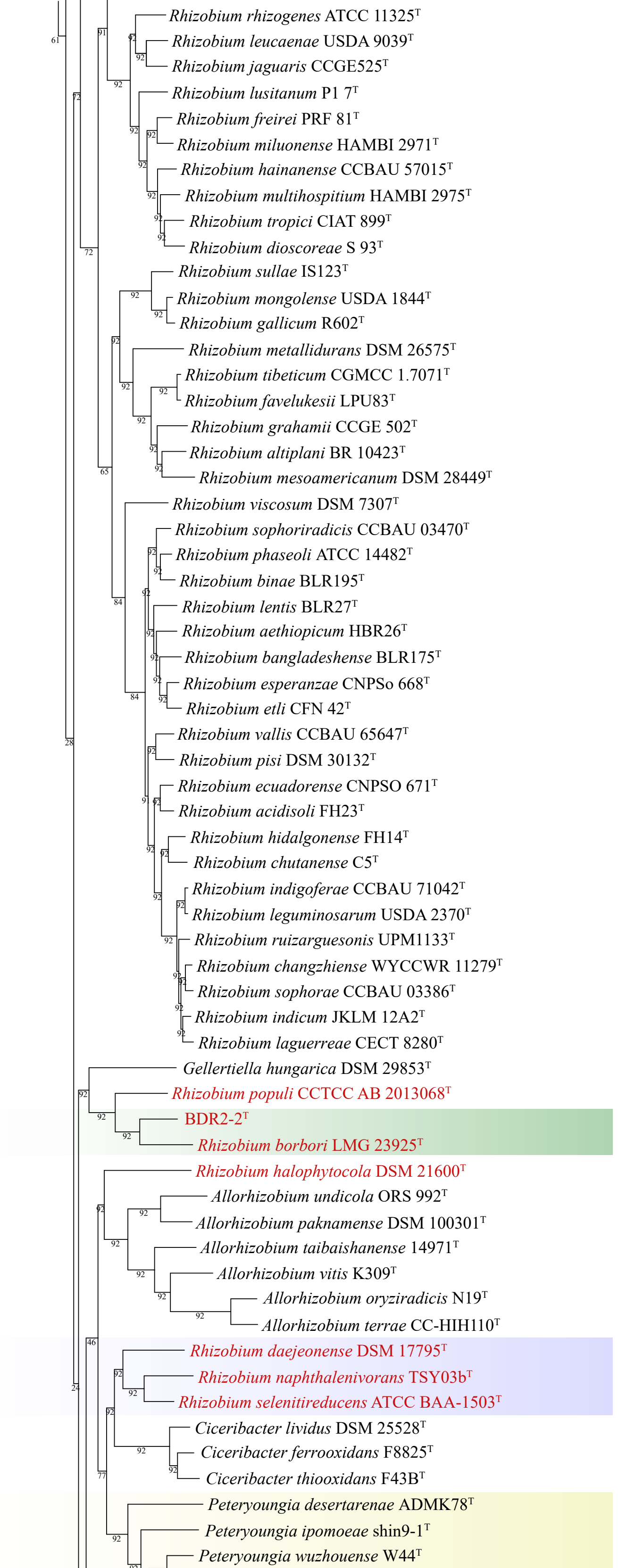
Rhizobium rhizogenes ATCC 11325T
Rhizobium leucaenae USDA 9039T
Rhizobium jaguaris CCGE525T
Rhizobium lusitanum P1 7T
Rhizobium freirei PRF 81T
Rhizobium miluonense HAMBI 2971T
Rhizobium hainanense CCBAU 57015T
Rhizobium multihospitium HAMBI 2975T
Rhizobium tropici CIAT 899T
Rhizobium dioscoreae S 93T
Rhizobium sullae IS123T
Rhizobium mongolense USDA 1844T
Rhizobium gallicum R602T
Rhizobium metallidurans DSM 26575T
Rhizobium tibeticum CGMCC 1.7071T
Rhizobium favelukesii LPU83T
Rhizobium grahamii CCGE 502T
Rhizobium altiplani BR 10423T
Rhizobium mesoamericanum DSM 28449T
Rhizobium viscosum DSM 7307T
Rhizobium sophoriradicis CCBAU 03470T
Rhizobium phaseoli ATCC 14482T
Rhizobium binae BLR195T
Rhizobium lentis BLR27T
Rhizobium aethiopicum HBR26T
Rhizobium bangladeshense BLR175T
Rhizobium esperanzae CNPSo 668T
Rhizobium etli CFN 42T
Rhizobium vallis CCBAU 65647T
Rhizobium pisi DSM 30132T
Rhizobium ecuadorense CNPSO 671T
Rhizobium acidisoli FH23T
Rhizobium hidalgonense FH14T
Rhizobium chutanense C5T
Rhizobium indigoferae CCBAU 71042T
Rhizobium leguminosarum USDA 2370T
Rhizobium ruizarguesonis UPM1133T
Rhizobium changzhiense WYCCWR 11279T
Rhizobium sophorae CCBAU 03386T
Rhizobium indicum JKLM 12A2T
Rhizobium laguerreae CECT 8280T
Gellertiella hungarica DSM 29853T
Rhizobium populi CCTCC AB 2013068T
BDR2-2T
Rhizobium borbori LMG 23925T
Rhizobium halophytocola DSM 21600T
Allorhizobium undicola ORS 992T
Allorhizobium paknamense DSM 100301T
Allorhizobium taibaishanense 14971T
Allorhizobium vitis K309T
Allorhizobium oryziradicis N19T
Allorhizobium terrae CC-HIH110T
Rhizobium daejeonense DSM 17795T
Rhizobium naphthalenivorans TSY03bT
Rhizobium selenitireducens ATCC BAA-1503T
Ciceribacter lividus DSM 25528T
Ciceribacter ferrooxidans F8825T
Ciceribacter thiooxidans F43BT
Peteryoungia desertarenae ADMK78T
Peteryoungia ipomoeae shin9-1T
Peteryoungia wuzhouense W44T

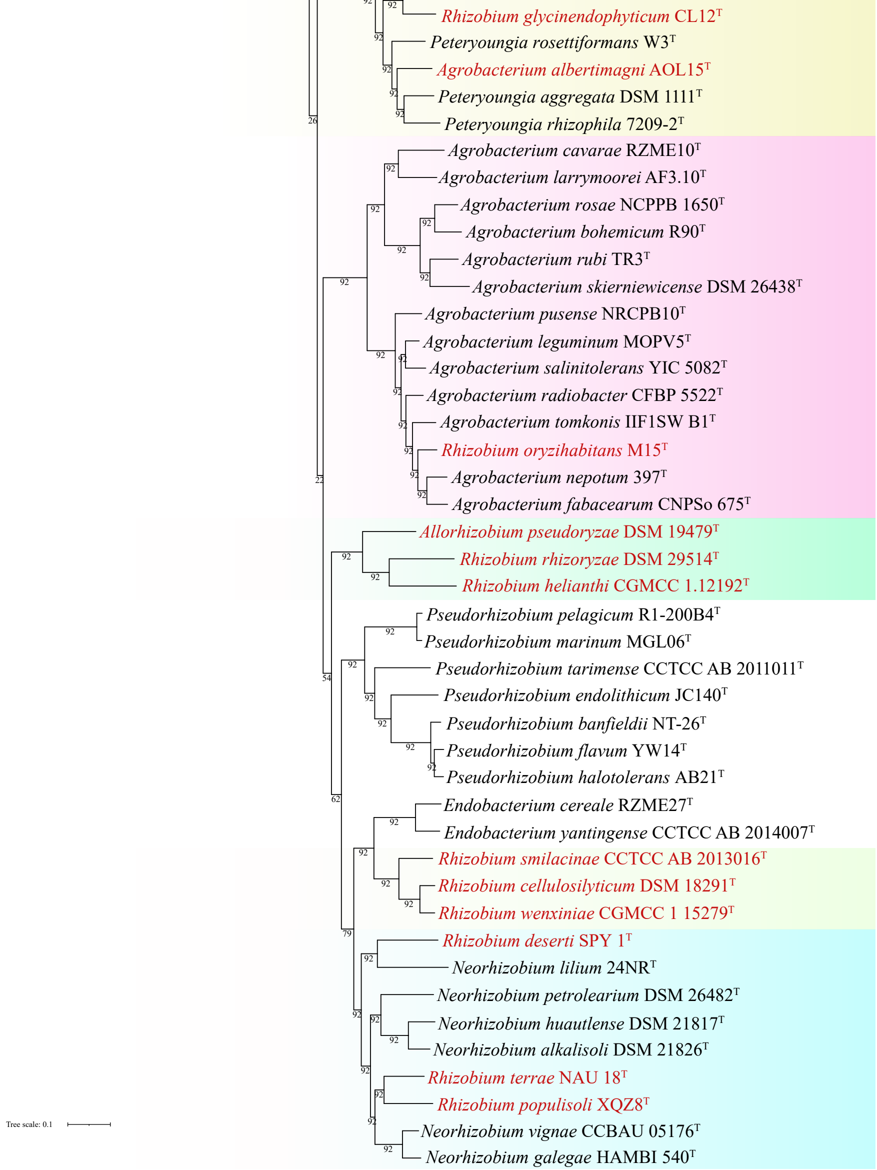

Figure S3. The UBCG phylogenetic tree among strains in family *Rhizobiaceae* based on 92 single copy core gene sequences. The strains in order *Caulobacterales* and family *Brucellaceae* were used as outgroup. The scale bar corresponds to 0.1

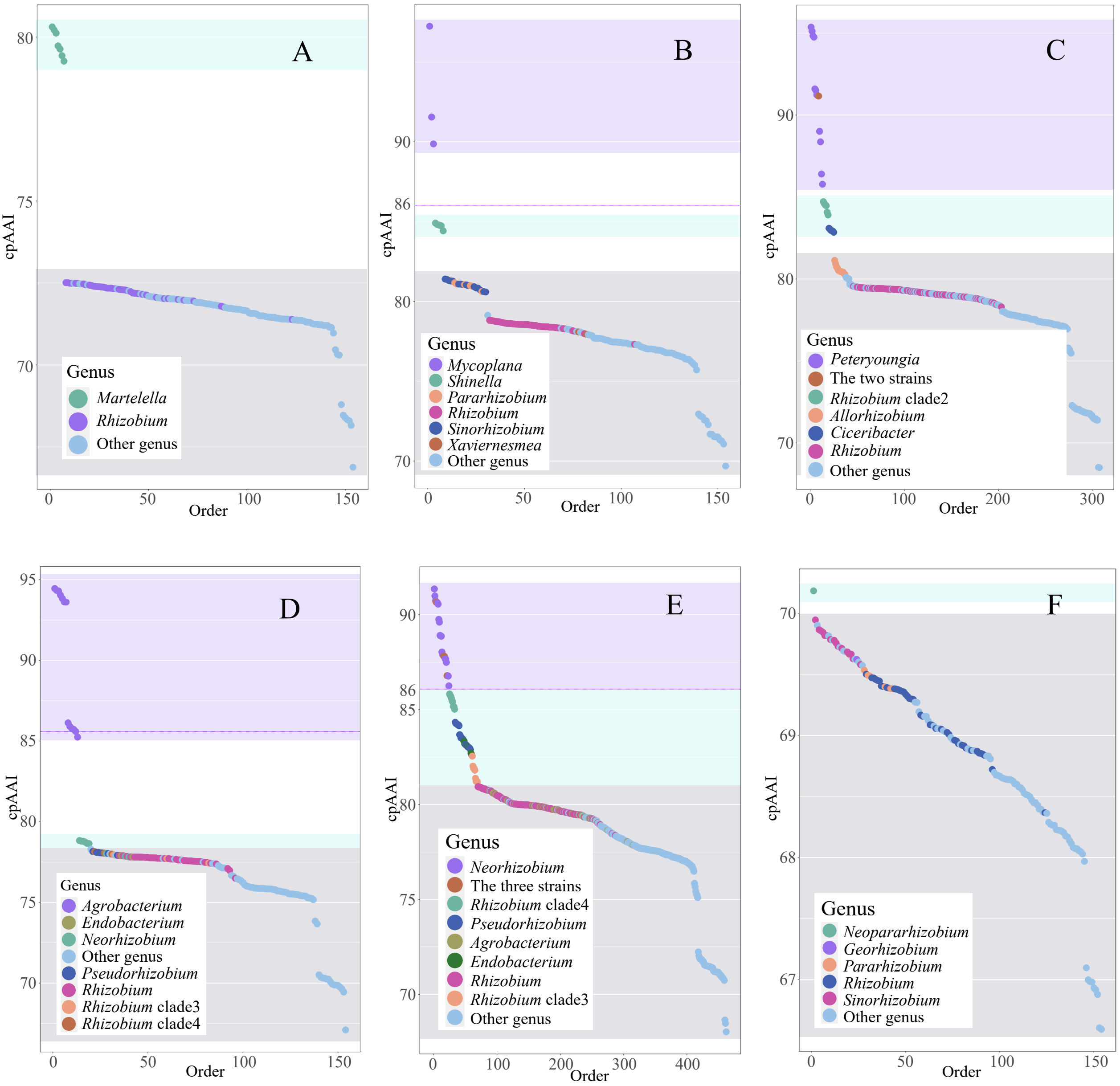

Figure S4. The rank order of pairwise cpAAI values of members within family *Rhizobiaceae*.
A, *Martelella alba* BGMRC 2036^{T} and other members;
B, *Rhizobium rhizolycopersici* DBTS2^{T} and other members;
C, values with *Rhizobium glycinendophyticum* CL12^{T} and *Agrobacterium albertimagni* AOL15^{T} and between the two strains and other members;
D, *Rhizobium oryzihabitans* M15^{T} and other members;
E, values with three strains (*Rhizobium deserti* SPY 1^{T}, *Rhizobium terrae* NAU 18^{T} and *Rhizobium populisoli* XQZ8^{T}) and between the three strains and other members;
F, *Pararhizobium mangrovi* BGMRC 6574^{T} and other members;

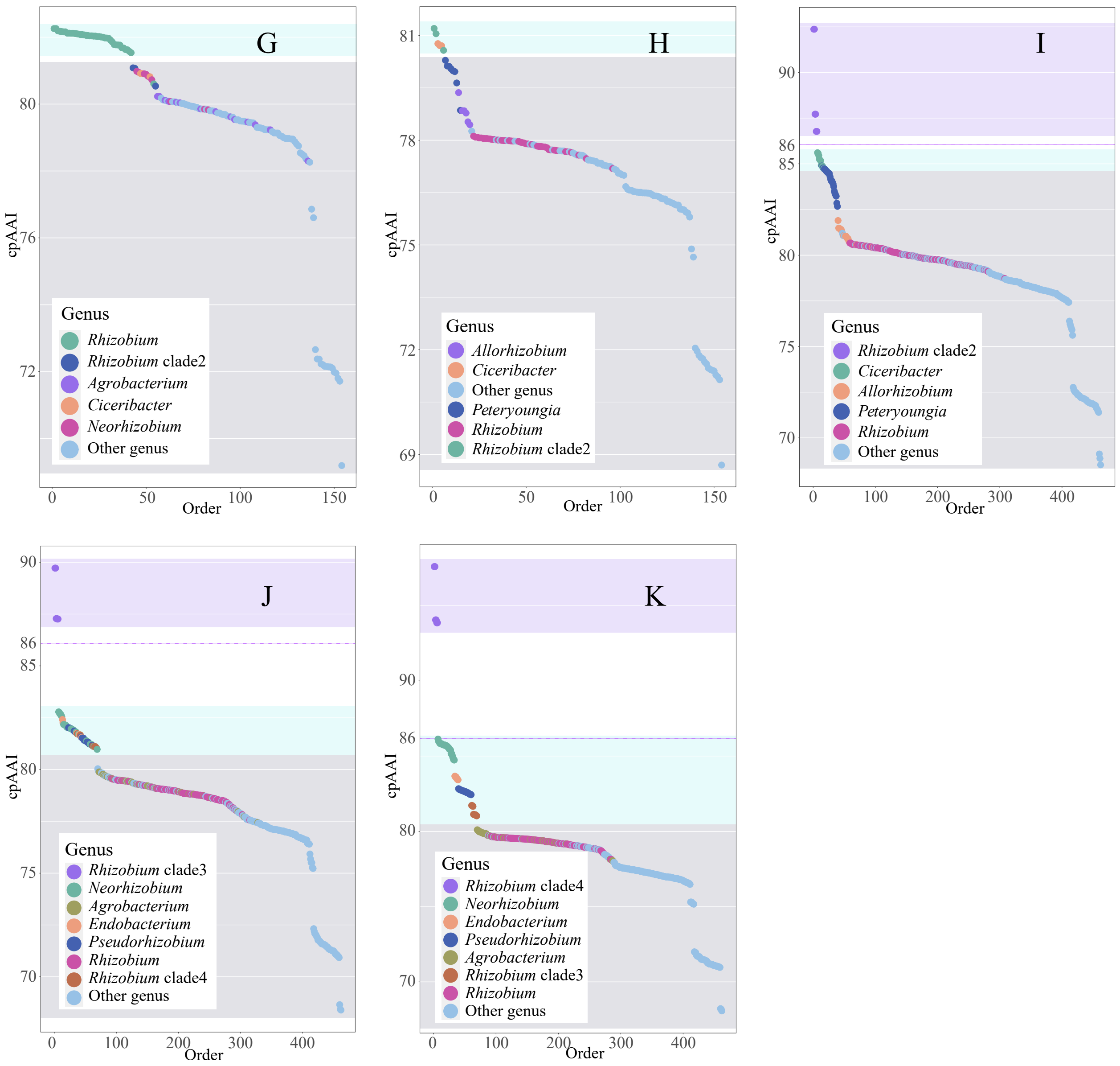

Figure S5. The rank order of pairwise cpAAI values of members within family *Rhizobiaceae*.
G, *Rhizobium album* NS-104^{T} and other members;
H, *Rhizobium halophytocola* DSM 21600^{T} and other members;
I, values within *Rhizobium* clade 2 and between *Rhizobium* clade 2 and other members;
J, values within *Rhizobium* clade 3 and between *Rhizobium* clade 3 and other members;
K, values within *Rhizobium* clade 4 and between *Rhizobium* clade 4 and other members.

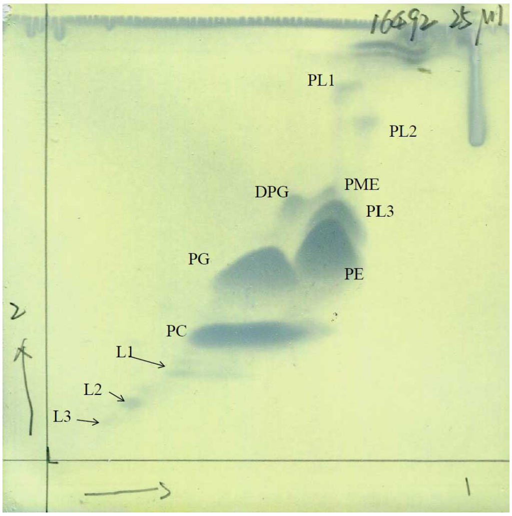

Figure S6. Polar lipid profiles of strain BDR2-2^T separated and detected by two-dimensional thin-layer chromatography and spraying with a molybdatophosphoric acid reagent, respectively. DPG, disphosphatidylglycerol; PE, phosphatidylethanol-amine; PG, phosphatidylglycerol; PL, phospholipid; AL, aminolipid; APL, unidentified

Table S2. Differential characteristics of the strain BDR2-2^T and closely related type strains.

Strains: 1, BDR2-2^T; 2, *Rhizobium borbori* DN316^T (data from Zhang et al. 2011, Rozahon et al. 2014); 3, *Rhizobium populi* K-38^T (data from Rozahon et al. 2014); +, Positive; -, negative; W, weakly positive.

Characteristic	1	2	3
pH range (optimum)	5.0-9.0 (7.0)	5.0-9.0 (6.5-7.0)	6.0-9.0 (7.5)
temperature range (optimum; °C)	10-41 (28)	4-37 (28)	25-37 (28)
NaCl tolerance (%, w/v)	0-2	0-0.5	0-3
Utilization of:			
D-Galactose, L-Fucose, D-Mannitol, D-Arabitol Citric Acid, D-Lactic Acid Methyl Ester	-	+	-
D-Salicin, Quinic Acid, Methyl Pyruvate, Propionic Acid, D-Aspartic Acid,	-	+	+
α-D-Lactose, Glycerol, L-Histidine, L-Pyroglutamic Acid, L-Serine, N-Acetyl-D-Glucosamine	+	-	-
myo-Inositol, D-Galacturonic Acid,	W	-	-
D-Gluconic Acid, D-Glucuronic Acid,			
Enzyme activities:			
Alkaline phosphatase, *β*-galactosidase,	+	-	-
esterase lipase (C8), lipase (C14)			
Esterase (C4), leucine arylamidase, valine arylamidase, cystine arylamidase, *β*-galactosidase	+	+	-
Trypsin, *α*-chymotrypsin	+	-	+
α-galactosidase, *α*-mannosidase, *α*-fucosidase	-	+	-
Acid production from:			
L-rhamnose, D-melibiose	+	-	-
L-arginine, L-ornithine, D-sorbitol	-	-	+
Predominant polar lipids	PE, PG, PC, DPG, PME	PE, PC, PG, DPG, PME	PE,PG, PC, PME, DPG,
G+C content (%)	64.5	61.3	64.9

Table. S3 Cellular fatty acid profiles of strain 16492^T and *Rhizobium borbori* DN316^T and *Rhizobium populi* K-38^T.

Fatty acid	1	2	3
Summed feature 8	61.9	55.2	57.2
$C_{19:0}$ cyclo $\omega 8c$	11.0	4.6	9.5
$C_{16:0}$	10.1	14.6	13.6
$C_{16:0}$ 3-OH	4.6	11.2	2.4
$C_{18:0}$	4.7	0.7	1.8
11-Methyl $C_{18:1}\omega 7c$	0.45	1.6	-
$C_{17:0}$ cyclo	-	1.1	-
$C_{14:0}$	0.29	0.8	0.4
$C_{18:0}$ 3-OH	-	0.1	0.6

1, 16492^T; 2, *Rhizobium borbori* DN316^T (data from Rozahon et al. 2014); 3, *Rhizobium populi* K-38^T (data from Rozahon et al. 2014); -, not detected, Summed feature 8 comprised $C_{18:1}\omega 7c$ and/or $C_{18:1}\omega 6c$.
